# Ecotypes of triple-negative breast cancer in response to chemotherapy

**DOI:** 10.1038/s41586-026-10469-9

**Published:** 2026-05-13

**Authors:** Yun Yan, Yiyun Lin, Tapsi Kumar, Shanshan Bai, Aatish Thennavan, Jianzhuo Li, Emi Sei, Tuan Tran, Min Hu, Mitchell Rao, Chenling Tang, Siyuan He, Anna Casasent, Elizabeth Ravenberg, Gaiane Margishvili Rauch, Alyson R. Clayborn, Debu Tripathy, Alastair Thompson, Bora Lim, Lei Huo, Stacy Moulder, Clinton Yam, Nicholas Navin

**Affiliations:** 1 Department of Systems Biology, UT MD Anderson Cancer Center, Houston, TX USA; 2 Graduate School of Biomedical Sciences, UT MD Anderson Cancer Center UTHealth Houston, Houston, TX USA; 3 Department of Genetics, UT MD Anderson Cancer Center, Houston, TX USA; 4 Department of Breast Medical Oncology, UT MD Anderson Cancer Center, Houston, TX USA; 5 Abdominal Imaging Department, UT MD Anderson Cancer Center, Houston, TX USA; 6 Department of Pathology, UT MD Anderson Cancer Center, Houston, TX USA; 7 Department of Surgery, Baylor Medical College, Houston, TX USA

**Keywords:** Breast cancer, Medical genomics, Tumour heterogeneity

## Abstract

Triple-negative breast cancer (TNBC) is an aggressive subtype that is frequently treated with chemotherapy, but only half of the patients respond well and have good clinical outcome^1,2^. Here we leveraged pretreatment tissue samples from treatment-naive patients with TNBC who received neoadjuvant chemotherapy and performed single-cell transcriptomic analysis of 427,857 cells from 101 patients and spatial transcriptomic analysis of 44 patients. We classified TNBC tumours into 4 patient-level subtypes (archetypes) using the cancer-cell gene expression and identified 13 metaprograms that reflect intra-tumoural heterogeneity at the single-cell level. The TNBC tumour microenvironment consisted of 49 immune and stromal cell states, many of which were reprogrammed relative to normal breast tissues. Furthermore, we identified eight distinct cellular communities (ecotypes) on the basis of the co-occurrences of cancer cells and tumour microenvironment cell types, and their spatial organization in tissues. In contrast to previous studies on T cells, our data show the importance of macrophage subtypes and cancer-cell metaprograms for interferon signalling, human leukocyte antigen expression and cell cycle activity that are associated with a good response to neoadjuvant chemotherapy. Collectively, this study provides new insights into the biology of untreated TNBC tumours and their association with chemotherapy response.

## Abstract (web summary)

Treatment data for triple-negative breast cancer show the importance of macrophage subtypes and cancer-cell metaprograms for interferon signalling, HLA expression and cell cycle activity that are associated with a good response to neoadjuvant chemotherapy.

## Main

Triple-negative breast cancer (TNBC) accounts for 10–20% of breast cancer cases and lacks oestrogen, progesterone and HER2 receptors, resulting in limited treatment options^3,4^. In early-stage TNBC, neoadjuvant chemotherapy (NAC) remains a foundational component of treatment, achieving pathological complete response (pCR) with improved survival in 40–50% of patients^1,2^. The current standard of care incorporates immune checkpoint inhibitors with chemotherapy, increasing pCR rates by 10–15%^2^. In metastatic TNBC, patients are treated with chemotherapy using antibody–drug conjugates^5^. Whereas chemotherapy is the pillar of treatment, it remains challenging to predict which patients with TNBC will respond and which patients should seek alternative therapeutic strategies.

TNBC shows inter-patient heterogeneity, which may explain variation in treatment responses. Bulk tissue gene expression-based studies have developed TNBC subtyping classifications, but they cannot resolve the diverse milieu of cell types in the tumour microenvironment (TME)^6–10^. Although computational deconvolution methods can be applied, they rely heavily on predefined marker genes^11–13^. TNBC also shows extensive intra-tumoural heterogeneity (ITH) due to genomic aberrations and diverse gene-expression programs across single cells within the same tumour^14,15^. Single-cell RNA sequencing (scRNA-seq) and spatial gene-expression profiling methods have been used to investigate TNBC, however, their relationship to therapeutic response remains unclear. Several studies have investigated ITH in TNBC, but lacked therapy response data^14,16–18^, whereas other studies have explored the clinical implications of ITH, but focused on either cancer cells^15^ or immune cells seperately^19–22^. The small cohort sizes (2 to 34 patients with TNBC) in these studies limited their statistical power to associate ITH with clinical outcomes. Spatial studies have also identified response-associated tissue patterns, but the small gene panels (fewer than 50 genes) or mixed cell resolution have made it challenging to resolve cell states at high granularity^22–27^.

Another key question in TNBC is how the cellular composition of tumours differs from normal breast tissues. Although many cell types including fibroblasts^28^, perivascular cells^29^ and immune cells^30,31^ are reprogrammed during tumour progression, their associations with therapy response in TNBC remain unclear. Furthermore, identifying the epithelial cell of origin that gives rise to TNBC has been challenging in bulk tissue studies^32^. Whereas basal and/or myoepithelial cells (basal) have been proposed as the potential cell of origin, other studies have implicated luminal secretory and/or progenitor cells (LumSec) and luminal hormone responsive cells (LumHR)^33^.

To address these questions, we performed scRNA-seq and spatial transcriptomic profiling of pretreatment breast core biopsy samples from treatment-naive patients with early-stage TNBC. We delineated cancer-cell metaprograms and TME cell states, and studied their co-occurrence and spatial organization across tissues. We further investigated their associations with NAC response and compared the TME composition in TNBC with normal breast tissues using the Human Breast Cell Atlas (HBCA) datasets^34^.

## Study design and TNBC cell types

**Fig. 1:**
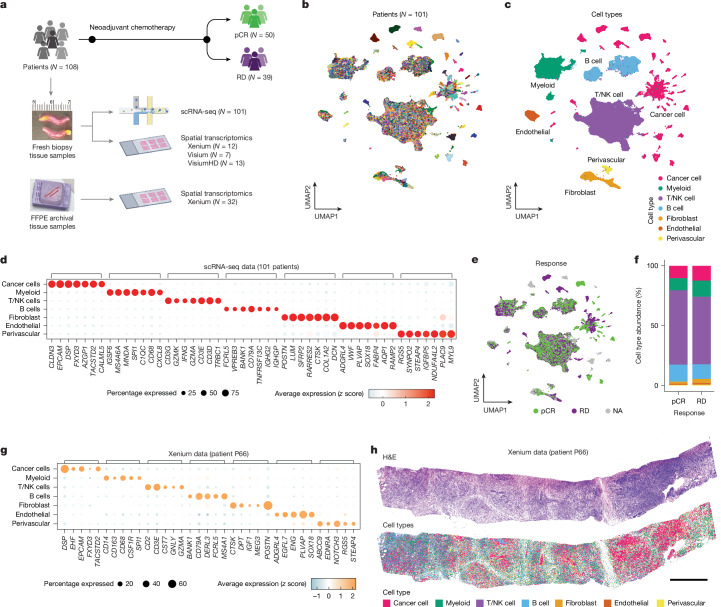
Study design and main cell types in TNBC. **a**, Experimental workflow for scRNA-seq and spatial transcriptomic profiling of patients with TNBC. **b**,**c**, Uniform manifold approximation and projection (UMAP) of the unintegrated scRNA-seq data from 427,857 cells coloured by the patients (**b**) and the main cell types (**c**). **d**, Dot plot showing the seven top DEGs in each cell type. **e**, UMAP of the unintegrated scRNA-seq data coloured by NAC response. NA, not applicable **f**, Bar plot showing the average cell-type percentages in patients with pCR and RD. **g**,**h**, Patient P66 Xenium data showing a dot plot of the five top DEGs in each cell type (**g**), and the haematoxylin and eosin (H&E) image with spatial locations of main cell types (**h**). Scale bar, 1,000 μm. Panel **a** created in BioRender; Yan, Y. https://biorender.com/zjf9vol (2026).

This study used pretreatment core biopsy samples from 108 treatment-naive patients with early-stage TNBC in the clinical trial ARTEMIS (NCT02276443)^35^ (Fig. 1a and Supplementary Table 1). Eligible patients were treated with NAC, which included doxorubicin and cyclophosphamide followed by paclitaxel-based therapy. NAC response was classified into pCR or residual disease (RD). In our samples, 50 patients experienced pCR and 39 patients had RD, whereas 19 patients did not have response data (Methods). Fresh tissue samples were collected within 1–2 hours and dissociated into viable cell suspensions for scRNA-seq. Frozen or formalin-fixed paraffin-embedded (FFPE) core biopsy samples were used for spatial transcriptomics experiments including Xenium, Visium and VisiumHD (10X Genomics) (Methods). Overall, 39 of 108 patients had matched scRNA-seq and spatial transcriptomic data.

**Extended Data Fig. 1:**
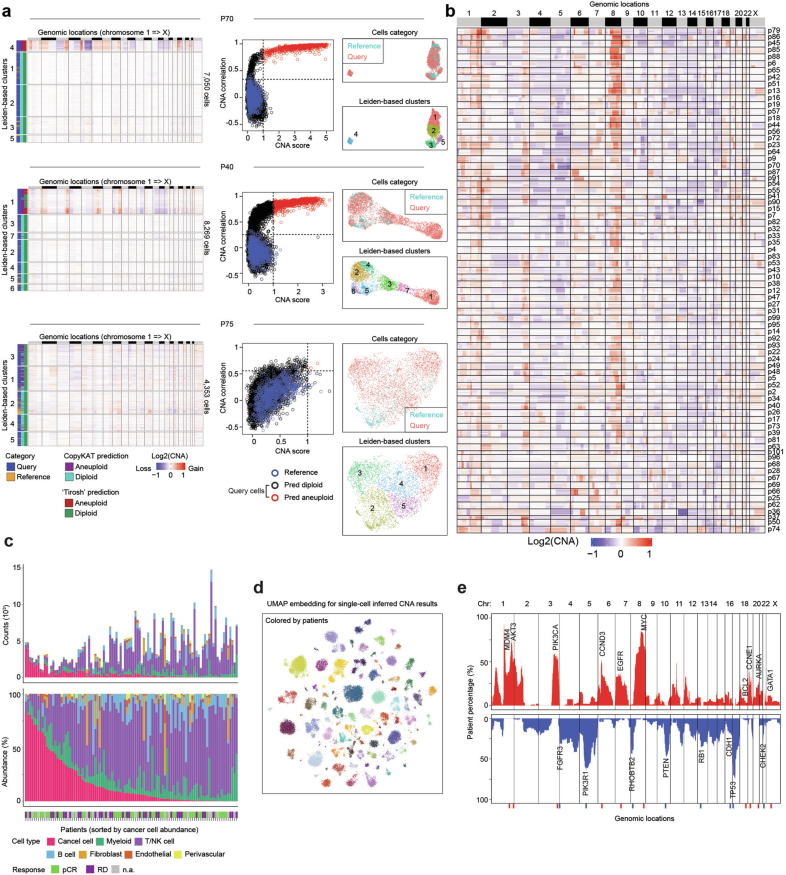
Copy number inference from scRNA-seq data, related to Fig. 1. **a**, Single-cell CNA profiles inferred from scRNA-seq data of three patient examples (P70, P40, and P75) with a heatmap showing CNA profiles (left), a scatter plot showing aneuploidy classification based on CNA scores and CNA correlations (middle), and a UMAP plot of the inferred CNA profiles (right). **b**, Heatmap showing the inferred single-cell CNA profiles of cancer cells grouped by patients. To facilitate valuation, patients with less than 50 cancer cells were excluded, and at most 100 cells were randomly selected per patient. **c**, Barplot showing cell type compositions measured in absolute cell count number (top) and percentages showing abundance (bottom) for each patient. **d**, UMAP of the inferred single-cell CNA profiles of 49,275 cancer cells colored by patients. **e**, Frequency plot of the consensus CNA events in TNBC patients.

The scRNA-seq data profiled an average of 7,265 cells with an s.e.m. of 373 per patient and an average sequencing depth of 75,172 reads (s.e.m. = 4,252) per cell, resulting in a total of 427,857 cells from 101 patients (mean = 4,236, s.e.m. = 248) after filtering (Supplementary Table 2 and Methods). Aneuploid cells were identified on the basis of the inferred copy number alterations (CNAs) from the scRNA-seq read counts using CopyKAT^36^ (Extended Data Fig. 1a,b and Methods). Unbiased clustering of the unintegrated data showed patient-specific clusters of cancer cells and patient-intermixed clusters, representing the main TME cell types (Fig. 1b,c and Methods). These TME clusters included three immune cell types (myeloid cells, T/natural killer (NK) cells and B cells) and three stromal cell types (fibroblasts, endothelial cells and perivascular cells) on the basis of the top differentially expressed genes (DEGs)^34^ (Fig. 1d and Methods). Cancer cells were found in 97 of 101 patients, with an average percentage of 17.12% (s.e.m. = 2.25%) and a range from 0% to 83.7% (Extended Data Fig. 1c). Although the cell-type composition was not significantly different between pCR and RD (*P* > 0.05, Wilcoxon), the different densities of many cell-type clusters across response groups prompted us to investigate the more granular differences in the cell-state compositions (Fig. 1e,f). Using the Xenium in situ RNA platform, we also measured expressions of roughly 5,000 targeted genes in 44 patients (Supplementary Tables 3 and 4 and Methods). The Xenium data identified the same main cell types as scRNA-seq and further revealed the spatial distributions of cells in tissues (Fig. 1g,h).

## Tumour archetypes and chemotherapy response

**Fig. 2:**
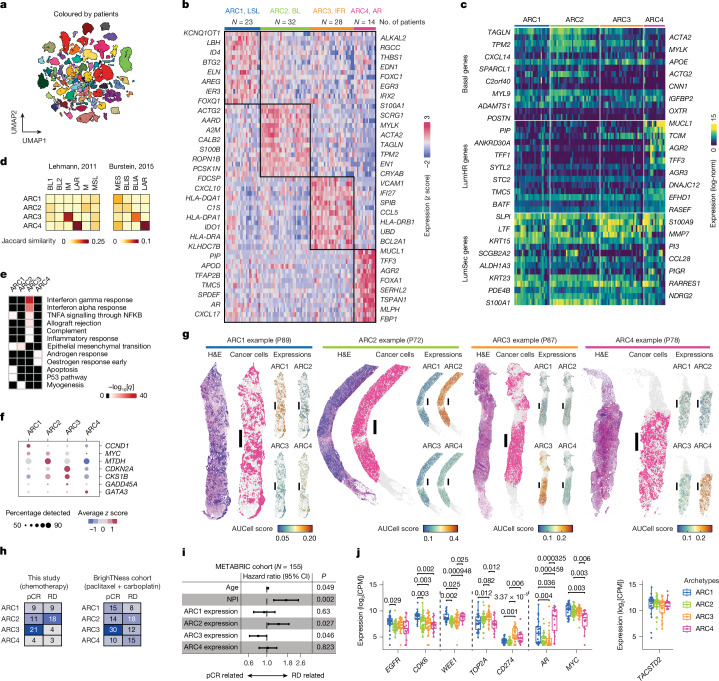
Patient-level archetypes of patients with TNBC identified from cancer cells. **a**, Unintegrated UMAP showing 49,275 single cancer cells that are coloured by patients (*N* = 97). **b**, Relative expressions of top 15 marker genes across patients grouped by archetypes. BL, basal-like; IFR, interferon responsive; LSL, luminal secretory-like. **c**, Relative expressions of the known normal breast epithelial lineage genes from HBCA in the archetypes. **d**, Jaccard similarity of DEGs between archetypes and previous TNBC subtypes from bulk expression studies^7,9^. BLIA, basal-like immune-activated; BLIS, basal-like immune-suppressed; IM, immunomodulatory; LAR, luminal AR; M, mesenchymal; MES, mesenchymal; MSL, mesenchymal stem-like. **e**, Gene enrichment of the archetype signatures against the MSigDB cancer hallmark database. **f**, Relative expression of the known cancer genes that were differently expressed across archetypes. **g**, Xenium data of four representative patients for archetypes showing the H&E images, the locations of cancer cells (red dots) and the overall expression scores of archetype gene signatures in cancer cells. **h**, Contingency table showing the number of patients grouped by archetype and therapy response in this study (left) and the BrighTNess cohort (right). **i**, Multivariate Cox proportional-hazards model testing the associations between the archetype gene signature expressions and the overall survival in the METABRIC cohort. NPI, Nottingham prognostic index. **j**, Normalized RNA expression of clinically targetable genes across archetypes. Group-wise comparisons per gene were conducted by Wilcoxon rank-sum test and *P* values were adjusted for false discovery rate. Only comparisons showing adjusted *P* values <0.05 are labelled. CPM, counts per million. Box plots show the median (centre line), interquartile range (box limits) and 1.5× the interquartile range (whiskers). Scale bars 1,000 µm.

**Extended Data Fig. 2:**
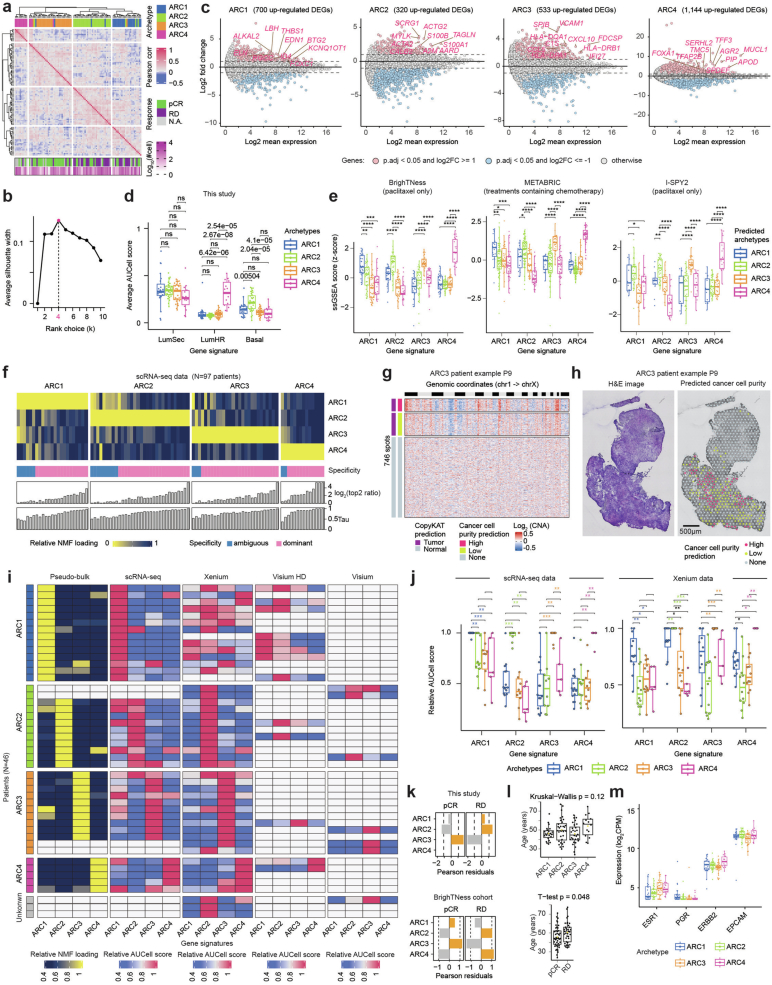
Archetype clustering and spatial transcriptomic analysis, related to Fig. 2. **a**, Patient-wise Pearson correlations based on the pseudo-bulk RNA-seq data of cancer cells. **b**, Average silhouette width as a function of the rank of NMF. **c**, MA-plot showing DEGs for each archetype compared with the other archetypes. **d**, Boxplot showing the average module scores of each epithelial lineage gene signature across archetypes. **e**, Boxplot showing the average ssGSEA scores of archetype gene signatures across the predicted archetypes in external cohorts. **f**, Archetype specificity in patients with heatmap showing the NMF loading matrix of archetypes among patients (top), the archetype specificity (middle), and barplot showing the Tau score and the signal ratio between the 2 top archetypes (lower). **g**, Heatmap showing CNAs profiles of spots inferred from the Visium data in P9. **h**, Visium data of P9 showing H&E staining and the predicted cancer cell purity. **i**, Heatmap showing the relative pseudo-bulk NMF loading matrix and the relative module score of the archetype gene signatures in the available scRNA-seq, Xenium, VisiumHD, and Visium data for each patient. **j**, Boxplot showing the relative module scores of archetype gene signatures using the genes overlapped in the Xenium gene panel. **k**, Pearson residuals of the Chi-squared test result of the contingency table of archetypes and responses. **l**, Boxplots showing patient age across archetypes and response groups. **m**, Boxplots showing normalized RNA expression of *ESR1*, *PGR*, *ERBB2* and *EPCAM* in the pseudo-bulk cancer cell data of patients across archetypes. CPM: counts per million UMIs. In **d**, **e**, and **j**, archetype-wise Wilcoxon rank-sum test was conducted for each gene signature with P-values adjusted by the Benjamini-Hochberg method. *: Adjusted *P* < 0.05; **: Adjusted *P* < 0.01; ***: Adjusted *P* < 0.001; ****: Adjusted *P* < 0.0001. Box plots show the median (center line), interquartile range (box limits), and 1.5x interquartile range (whiskers).

Unbiased clustering of 49,275 cancer cells from 97 patients identified many patient-specific clusters, consistent with other single-cell studies^37–39^ (Fig. 2a). The extensive inter-patient heterogeneity in gene expression reflected different CNA profiles of each patient, despite having some recurrent CNA events (for example, gains of 1q, 3q, 7p and 8q, and losses of 5q, 8p, 10q and 17p)^40,41^ (Extended Data Fig. 1b,d,e). Several TNBC subtyping frameworks have been developed^6^ but were derived from bulk tissue sequencing methods. These signatures are confounded by TME cell types and variations in tumour purity, making it unclear whether subtypes such as the immunomodulatory groups reflected the gene expressions of cancer cells or immune cells^9,10^. To address this limitation, we focused on the pure cancer-cell-specific scRNA-seq data, which were free from contamination from non-epithelial cells. Briefly, we aggregated the scRNA-seq data of cancer cells in each patient and performed a pseudo-bulk analysis on all patients (Methods). This analysis identified four main cancer-intrinsic expression programs at the patient level, which we termed ‘archetypes’: ARC1, ARC2, ARC3 and ARC4 (Extended Data Fig. 2a,b). On the basis of their gene-expression signatures, we annotated ARC1 as LumSec-like, ARC2 as basal-like, ARC3 as interferon responsive and ARC4 as androgen receptor (AR)-enriched, as explained in more detail below (Fig. 2b, Extended Data Fig. 2c and Supplementary Tables 5 and 6).

We inferred the epithelial lineage associated with archetypes on the basis of expression of the LumSec, LumHR and basal and/or myoepithelial marker genes^34^. Although all archetypes showed high expression of LumSec markers (for example, *KRT15*, *SLPI*,* KRT23*), ARC2 also showed high expression of basal markers (for example, *TAGLN*, *ACTA2*) and ARC4 expressed LumHR markers (for example, *PIP*,* ANKRD30A*) (Fig. 2c and Extended Data Fig. 2d). These findings indicate LumSec as the potential cell of origin for TNBC, whereas extra markers of the basal (ARC2) and LumHR (ARC4) lineages may have emerged during tumour progression. Compared with previous TNBC subtypes^7,9^, ARC1 overlapped with the mesenchymal subtypes, whereas ARC2 corresponded to the basal subtype (Fig. 2d and Methods). ARC3 strongly aligned with the immunomodulatory subtype, indicating that this subtype reflects the gene-expression programs of cancer cells rather than the adjacent immune cells. ARC4 matched strongly to the luminal AR subtype (Fig. 2d).

We further annotated archetypes using the cancer hallmark gene sets (Fig. 2e and Methods). ARC1 was enriched for apoptosis, *TNFA* signalling by means of *NFKB*, *TP53* signalling and epithelial-to-mesenchymal transition (EMT). ARC2 showed enrichment for EMT and myogenesis, consistent with its basal lineage. ARC3 was enriched for interferon-γ or interferon-α response, and inflammatory response pathways, aligning with the high expression of many immune-signalling genes (*IFI27*,* CXCL10* and *HLA-DRA*) (Fig. 2b). ARC4 was enriched for androgen response and oestrogen early response pathways, consistent with its association with luminal AR. To facilitate developing biomarkers for archetypes, we investigated the DEGs that were associated with breast cancer^41^ (Fig. 2f). ARC1 was enriched for *CCND1* and *MYC*, and ARC2 showed higher expression of *MTDH*^42^. ARC3 highly expressed *CDKN2A*, *CKS1B* and *GADD45A*, related to cell cycle checkpoints and senescence^43^, whereas ARC4 had high expression of *GATA3*.

In three external cohorts (METABRIC^40^, BrighTNess^44^ and I-SPY2^45^) with bulk expression data available, we found that the patients with TNBC were well stratified into subgroups according to the archetype gene signatures (Extended Data Fig. 2e and Methods). Although 20.6% (20 out of 97) of patients in our cohort showed comparable expression in two or more archetypes, most (79.4%, 77 out of 97) were dominated by a single archetype (Extended Data Fig. 2f and Methods). To perform in situ validation, we applied three spatial transcriptomic technologies (Xenium, VisiumHD and Visium) to samples from 46 patients with the matched scRNA-seq data, showing that archetype classification was concordant across technologies (Fig. 2g and Extended Data Fig. 2g,h). Similarly, 80% (37 out of 46) of patients were dominated by one archetype in the spatial transcriptomic data (Extended Data Fig. 2i,j and Methods).

Archetype classification was significantly associated between and NAC response in our cohort (*P* = 7.1 × 10^−3^, chi-squared test) and in the BrighTNess cohort^44^ (*P* = 0.024, chi-squared test). ARC2 was associated with RD, whereas ARC3 was associated with pCR (Fig. 2h and Extended Data Fig. 2k). In the METABRIC cohort^40^, ARC2 was significantly associated with shorter overall survival and ARC3 was significantly correlated with longer overall survival (*P* < 0.05, Wald test) (Fig. 2i and Methods). Although the patients showing pCR in our cohort were younger (*P* = 0.048, *t*-test), consistent with previous clinical findings^46^, age did not differ significantly across archetypes (*P* = 0.12, Kruskal–Wallis test), indicating that the archetype classification was independent of age (Extended Data Fig. 2l). To explore potential targeted therapies for archetypes, we compared the expression of clinically druggable genes across archetypes (Fig. 2j). ARC1 had the highest expression of *EGFR* and *CDK6*, whereas ARC2 expressed lower levels of the G2-phase checkpoint gene *WEE1*. ARC3 had the highest expression of *TOP2A* and *CD274*, and ARC4 had the highest expression of *AR*. Notably, all archetypes expressed *TROP2* at a high level comparable to *EPCAM* (Fig. 2j and Extended Data Fig. 2m).

## Tumour metaprograms and NAC response

**Fig. 3:**
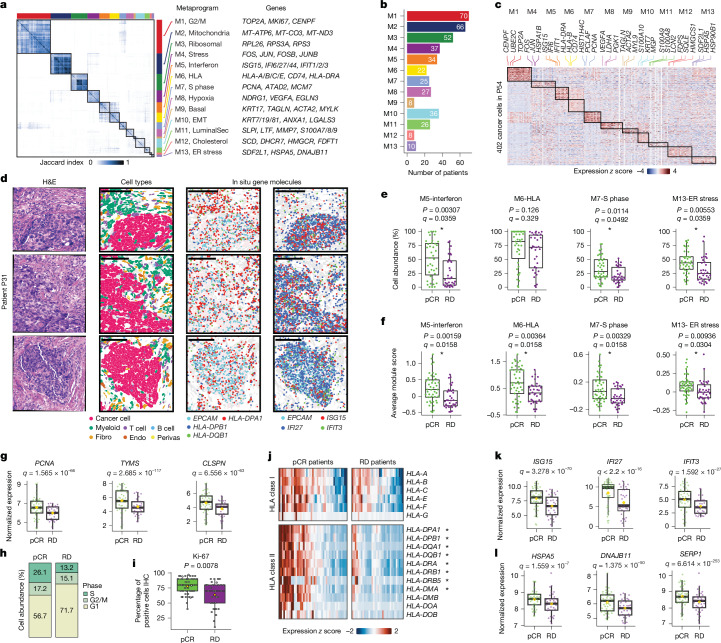
Intra-tumoural metaprograms of cancer cells. **a**, Jaccard index between all ITH gene-expression programs that were grouped by metaprograms. **b**, Bar plot showing the number of patients in whom the metaprograms were detected. **c**, Relative expressions of the metaprogram marker genes across single cells in a patient example (P54). **d**, Xenium data in a patient example (P31) showing the H&E images, the locations of the main cell types and the in situ expressions of *EPCAM* with the marker genes of the M6-HLA and M5-interferon metaprograms. **e**,**f**, Box plots showing the average cellular abundance (**e**) and the average module score (**f**) of metaprograms in the comparison of pCR and RD. **g**, Box plot showing the normalized pseudo-bulk RNA expression of three representative genes of M7-S. **h**, Bar plot showing the average cell abundance of the computationally estimated cell cycling phases in patients with pCR and RD. **i**, Box plot comparing the percentage of cells with positive immunohistochemistry staining for Ki-67 (Wilcoxon test exact *P* value). **j**, Heatmap showing the relative gene expression of the major histocompatibility complex class I and class II genes across patients. **k**, Box plot showing the normalized pseudo-bulk RNA expression of three representative interferon-related genes of M5-interferon. **l**, Box plot showing the normalized pseudo-bulk RNA expression of three representative genes of M13-ER stress. In **e**,**f**, Wilcoxon rank-sum tests were conducted with *P* values adjusted by the Benjamini–Hochberg method. In **g**,**j**–**l**, the Wald test was conducted by DESeq2 with *P* values adjusted by the Benjamini–Hochberg method. Asterisks indicate adjusted *P* < 0.05. Box plots show the median (centre line), interquartile range (box limits) and 1.5× the interquartile range (whiskers). Scale bars, 100 μm.

**Extended Data Fig. 3:**
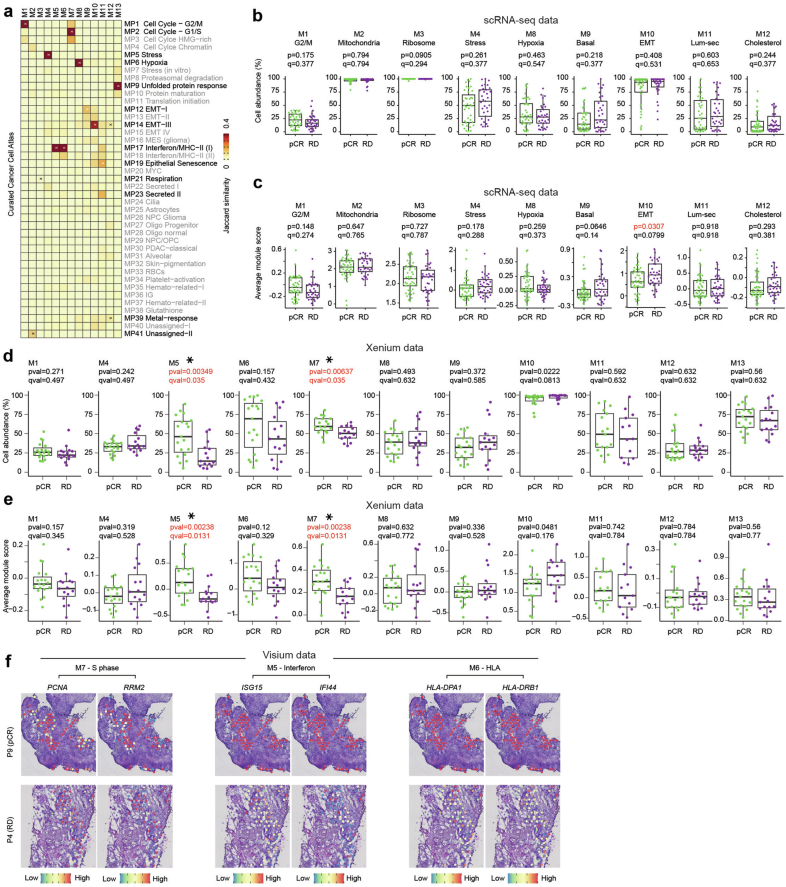
Cancer cell metaprograms and chemotherapy response, related to Fig. 3. **a**, Jaccard similarity between the cancer cell metaprograms identified in this study compared to the programs reported in the pan-cancer Curated Cancer Cell Atlas. A cross symbol denotes the most similar match. **b**,**c**, Boxplots showing the cellular percentage (**b**) and average module score (**c**) of the metaprograms in the scRNA-seq data of cancer cells in a comparison of response groups. **d**,**e**, Boxplots showing the cellular percentage (**d**) and average module score (**e**) of the metaprograms in the Xenium data of cancer cells in a comparison of the response groups. **f**, Visium data showing representative marker genes of the metaprograms in two patients. In **b**-**e**, Wilcoxon rank-sum test was conducted with P-values adjusted by the Benjamini-Hochberg method, with significant values colored in red. Box plots show the median (center line), interquartile range (box limits), and 1.5x interquartile range (whiskers).

To resolve ITH among cancer cells, we performed metaprogram analysis to cancer cells in each patient as previously described^47^ (Methods). We identified 13 recurrent metaprograms across patients, including 2 cell cycle-related metaprograms M1-G2/M and M7-S (S phase), 2 energy metabolism-related metaprograms M2-mito (mitochondrial) and M3-ribo (ribosomal), 3 stress-related metaprograms M4-stress, M8-hypoxia and M13-ER stress (endoplasmic reticulum stress), 2 immune-signalling-related metaprograms M5-interferon (interferon signalling) and M6-HLA (human leukocyte antigen), and 4 metaprograms M9-basal, M11-LumSec, M10-EMT and M12-cholesterol (cholesterol synthesis) that reflected epithelial lineages (Fig. 3a,b and Supplementary Table 7). These metaprograms reflected the diverse expression programs of cancer cells that co-occur within a tumour mass (Fig. 3c). The Xenium data confirmed the cancer-cell-intrinsic expression of HLA class II genes (for example, *HLA-DPA1/DPB1/DQB1*) and interferon signalling genes (for example, *ISG15*,* IFI27*,* IFIT3*) (Fig. 3d). Many metaprograms were consistent with the pan-cancer signatures from the Curated Cancer Cell Atlas^47^ (Extended Data Fig. 3a).

We next investigated associations between metaprograms and NAC response (Fig. 3e,f and Methods). M5-interferon, M7-S and M13-ER stress showed significantly higher cell frequencies and signature scores in pCR compared with RD (*q* < 0.05, Wilcoxon), indicating a compositional difference in cancer cells (Fig. 3e,f). By contrast, M6-HLA only showed a significantly higher signature score in pCR (*q* < 0.05, Wilcoxon) without showing different frequencies (*q* > 0.05, Wilcoxon), suggesting elevated expression within a subset of cancer cells rather than an increase of cellular abundance (Fig. 3e,f). The remaining metaprograms had no significant differences between pCR and RD (*P* or *q* > 0.05, Wilcoxon) (Extended Data Fig. 3b,c). Xenium and Visium data confirmed the significantly increased M5-interferon and M7-S programs in pCR (*q* < 0.05, Wilcoxon) (Extended Data Fig. 3d–f).

We investigated the four response-associated metaprograms in more detail. M7-S showed increased S-phase gene expression in pCR tumours (Fig. 3g). The cell cycle phase inference showed enrichment of S and G2/M phases in pCR and the non-cycling cells of G1/G0 in RD, consistent with the higher Ki-67 (*MKI67*) staining in pCR (*P* = 0.0078, Wilcoxon) (Fig. 3h,i and Methods). For M6-HLA, HLA class I genes showed similar expressions across NAC response groups, whereas HLA class II genes showed higher expression in pCR (Fig. 3j). Although HLA genes are typically expressed by professional antigen-presenting cells, cancer cells can also express HLA class II genes, reflecting immune cell mimicry in tumours, as emerging studies have reported^18,47,48^. M5-interferon showed higher interferon-induced gene expression in pCR (Fig. 3k). M13-ER stress included heat-shock and ER-stress genes that were upregulated in pCR (Fig. 3l).

## Immune cell states and NAC response

**Fig. 4:**
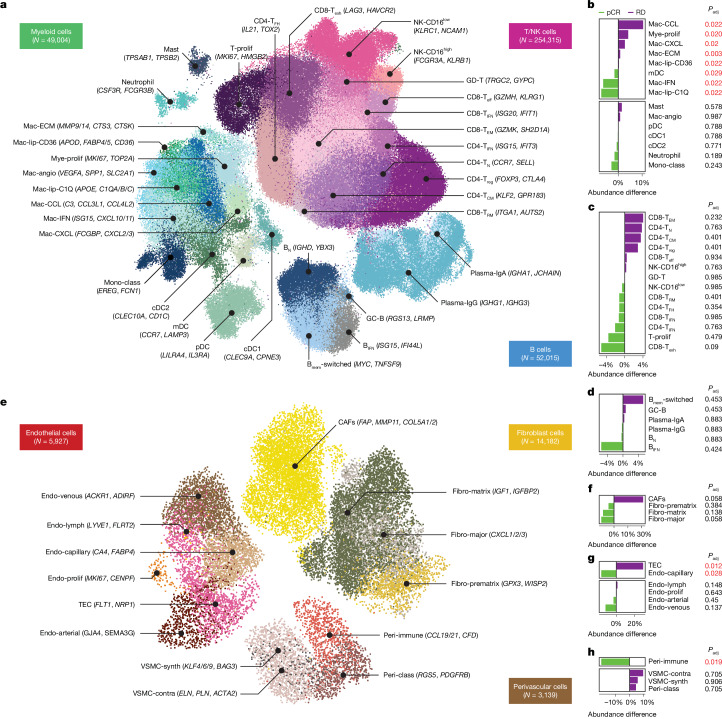
Immune and stromal cell states and their associations with NAC response. **a**, UMAP plot of the unintegrated scRNA-seq data showing 355,334 immune cells from 3 main cell types: myeloid cells, T/NK cells and B cells, with cell-state names and marker genes labelled. B_mem_-switched, switched-memory B cell; B_N_, naive B cell; GC-B, germinal centre B cell; mDC, mature dendritic cell; pDC, plasmacytoid dendritic cell. **b**–**d**, Cellular percentage difference in cell states of myeloid cells (**b**), T/NK cells (**c**) and B cells (**d**) in a comparison of patients with pCR and RD. **e**, UMAP plot of the unintegrated scRNA-seq data showing 23,248 stromal cells from 3 main cell types: fibroblast, endothelial cells and perivascular cells, with cell-state names and marker genes labelled. **f**–**h**, Cellular percentage difference in cell states of fibroblast cells (**f**), endothelial cells (**g**) and perivascular cells (**h**) in a comparison of patients with pCR and RD. In **b**–**d** and **f**–**h**, Wilcoxon rank-sum test was conducted with *P* values adjusted by the Benjamini–Hochberg method. Statistically significant values are coloured in red.

**Extended Data Fig. 4:**
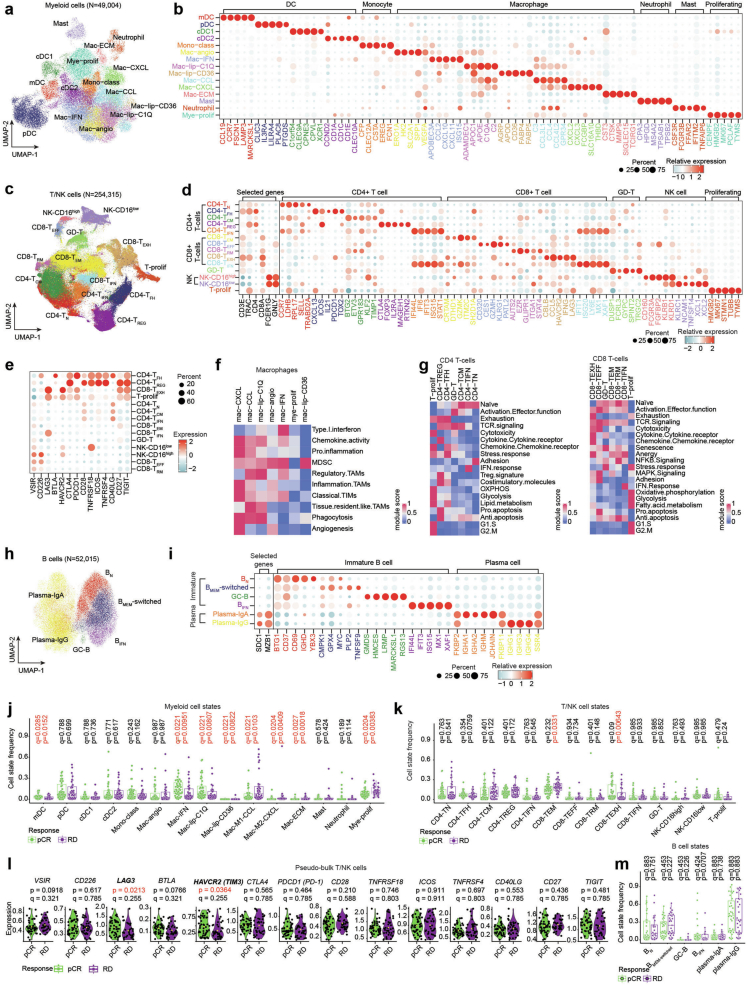
Immune cell states and their associations with chemotherapy response, related to Fig. 4. **a**, UMAP plot of the unintegrated scRNA-seq data showing N = 49,004 myeloid single cells colored by cell states. **b**, Dotplot showing the relative expression of marker genes of the myeloid cell states. **c**, UMAP plot of the unintegrated scRNA-seq data showing N = 254,315 T/NK single cells colored cell states. **d**, Dotplot showing the relative expression of marker genes for the T/NK cell states. **e**, Dotplot of the immune checkpoint genes across the T/NK cell states. **f**,**g**, Heatmap showing the average module score of the known functional gene signatures across the cell states of myeloid cells (**f**) and T-cells (**g**). **h**, UMAP plot of the unintegrated scRNA-seq data showing N = 52,015 B cells colored by the B-cell states. **i**, Dotplot showing the relative expression of marker genes of the B-cell states. **j**,**k**, Boxplot showing the cellular frequencies of the cell states of myeloid cells (**j**) and T/NK cells (**k**) in a comparison of pCR and RD. **l**, Normalized expression of the known immune checkpoint genes in T/NK cells in a comparison of the pCR and RD patients. Yellow dots denote the mean values. **m**, Cellular frequency of the cell states of B-cells in a comparison of the pCR and RD patients. In **j**-**m**, Wilcoxon rank-sum test was conducted with P-values adjusted by the Benjamini-Hochberg method. Box plots show the median (center line), interquartile range (box limits), and 1.5x interquartile range (whiskers).

The scRNA-seq data included three immune cell compartments: myeloid cells (*N* = 49,004), T/NK cells (*N* = 254,315) and B cells (*N* = 52,015) (Fig. 4a). Each compartment was further clustered to identify cell states on the basis of DEGs (Supplementary Table 8). The myeloid cells comprised 15 cell states spanning dendritic cells, monocytes, macrophages, neutrophils, mast cells and proliferating myeloid cells (Fig. 4a and Extended Data Fig. 4a,b). Dendritic cells included mature dendritic cells, plasmacytoid dendritic cells and conventional dendritic cells of cDC1 (*CLEC9A*, *CPNE3*) and cDC2 (*CLEC10A*, *CD1C*). Macrophages contained chemokine-related Mac-CCL (*CCL3L1*, *CCL4L2*) and Mac-CXCL (*FCGBP*, *CXCL2/3/8*) cells, lipid-related Mac-lip-CD36 (*FABP5*, *CD36*) and Mac-lip-C1q (*APOE*, *C1QA/B/C*) cells, angiogenesis-related Mac-angio (*VEGFA*, *SPP1*), extracellular matrix remodelling-related Mac-ECM (*CTSK*, *CST3*, *MMP9*) and interferon-responsive Mac-IFN cells (*ISG15*, *IFIT1/2/3*). The T/NK cells comprised 12 T cells and 2 NK cell states (Fig. 4a and Extended Data Fig. 4c,d). CD4 T cells included naive (CD4-T_N_), follicular helper (CD4-T_FH_), central memory (CD4-T_CM_), regulatory (CD4-T_reg_) and interferon-responsive (CD4-T_IFN_) cells. CD8 T cells included effector memory (CD8-T_EM_), effector (CD8-T_eff_), resident memory (CD8-T_RM_), exhausted (CD8-T_exh_) and interferon-responsive (CD8-T_IFN_) cells. γδ T cells (*TRGC2*) and proliferative T cells were also identified. NK cells consisted of NK-CD16^high^ and NK-CD16^low^ populations. Immune checkpoint genes were enriched in CD4-T_reg_, CD4-T_FH_ and CD8-T_exh_ cells (Extended Data Fig. 4e). Our macrophage and T cell annotations were supported by previously reported gene signatures^49–51^ (Extended Data Fig. 4f,g). B cells comprised six cell states: naive, switched-memory, germinal centre (*RGS13*) and interferon-responsive (B_IFN_) cells, and plasma cells expressing IgA or IgG (Fig. 4a and Extended Data Fig. 4h,i).

**Extended Data Fig. 5:**
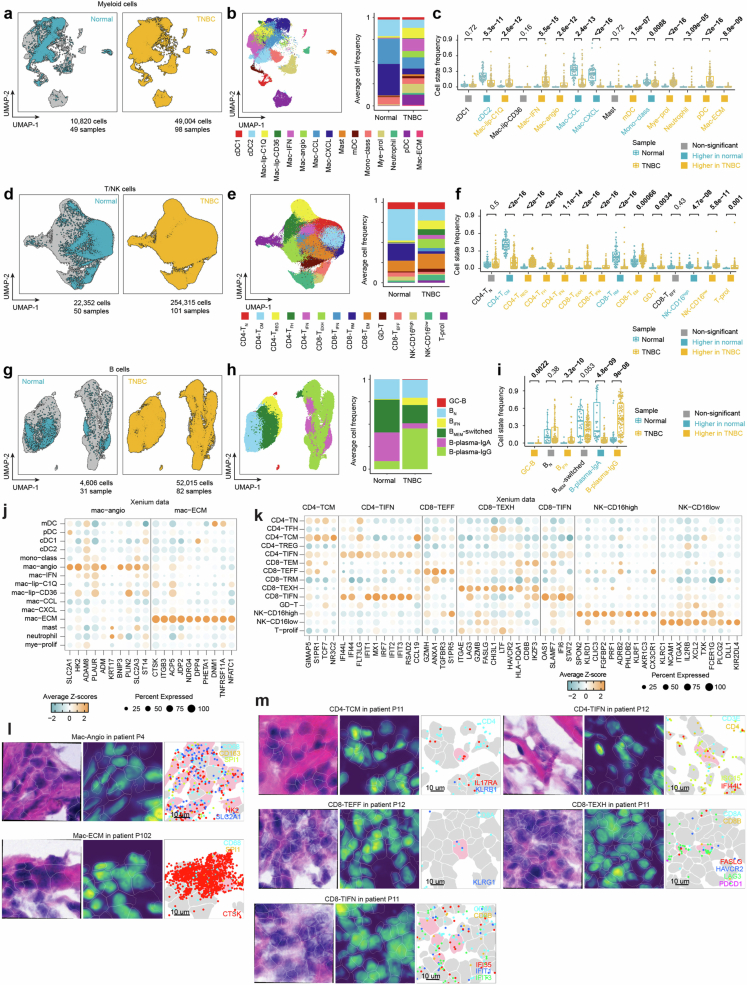
Immune cell state differences between normal breast and TNBC, related to Fig. 4. **a**, UMAP plot showing the single cells of myeloid cells in the integrated data of HBCA and TNBC. **b**, UMAP plot of the myeloid cell states and stacked barplot, showing the averaged cell state frequencies between HBCA and TNBC. **c**, Boxplot comparing the cellular frequency of myeloid cell states between HBCA and TNBC. **d**, UMAP plot showing the single cells of T/NK cells in the integrated data of HBCA and TNBC. **e**, UMAP plot of the T/NK cell states and stacked barplot showing the averaged cell state frequencies between HBCA and TNBC. **f**, Boxplot comparing the cellular frequency of T/NK cell states between HBCA and TNBC. **g**, UMAP plot showing the single cells of B-cells in the integrated data of HBCA and TNBC. **h**, UMAP plot of the B-cell states and stacked barplot showing the averaged cell state frequencies between HBCA and TNBC. **i**, Boxplot comparing the cellular frequency of B-cell states between HBCA and TNBC. **j**,**k**, Dotplot showing the relative expression of marker genes for 2 myeloid cell states (**j**) and 7 T/NK cell states (**k**) in the Xenium data. **l**,**m**, Xenium data showing in-situ expressions of marker genes for 2 macrophage cell states (**l**) and 5 T-cell states (**m**). In **c**,**f**, and **i**, Wilcoxon rank-sum test was conducted with P-values adjusted by the Benjamini-Hochberg method. Box plots show the median (center line), interquartile range (box limits), and 1.5x interquartile range (whiskers).

Integration with HBCA revealed stark differences in immune composition between TNBC and normal breast tissues from disease-free individuals (Methods). Most immune cell states significantly changed (*q* < 0.05, Wilcoxon), including 12 out of 15 myeloid cell, 12 out of 14 T/NK cell and 4 out of 6 B cell states, indicating extensive immune cell changes during tumour progression (Extended Data Fig. 5a–i). Notably, two macrophage cell states (Mac-angio and Mac-ECM) and 7 T/NK cell states (CD4-T_CM_, CD4-T_IFN_, CD8-T_eff_, CD8-T_exh_, CD8-T_IFN_, NK-CD16^high^, NK-CD16^low^) were specific to TNBC and were validated using Xenium (Extended Data Fig. 5j–m).

To compare immune composition between pCR and RD tumours, we analysed cell-state abundances within each cell type. Myeloid cells showed the most significant differences (*q *< 0.05, Wilcoxon). Mac-IFN and Mac-lip-C1Q cells were enriched in pCR, whereas Mac-CCL, Mye-prolif, Mac-CXCL and Mac-ECM cells were enriched in RD (Fig. 4b and Extended Data Fig. 4j). Compared with previous work studying lymphocytes at cell-type level^52^, we found that CD8-T_exh_ was increased in pCR and CD8-T_EM_ was higher in RD (*P* < 0.05, Wilcoxon), although these differences were not statistically significant after correction (*q* > 0.05, Wilcoxon) (Fig. 4c and Extended Data Fig. 4k). Immune checkpoint genes *LAG3* and *HAVCR2* (encoding TIM-3) that were enriched in CD8-T_exh_ cells showed higher expression in the pseudo-bulk data of pCR tumours, providing potential immune checkpoint inhibitor targets (Extended Data Fig. 4e,l). No B cell states differed between pCR and RD (*q* > 0.05, Wilcoxon) (Fig. 4d and Extended Data Fig. 4m).

## Stromal cell states and NAC response

**Extended Data Fig. 6:**
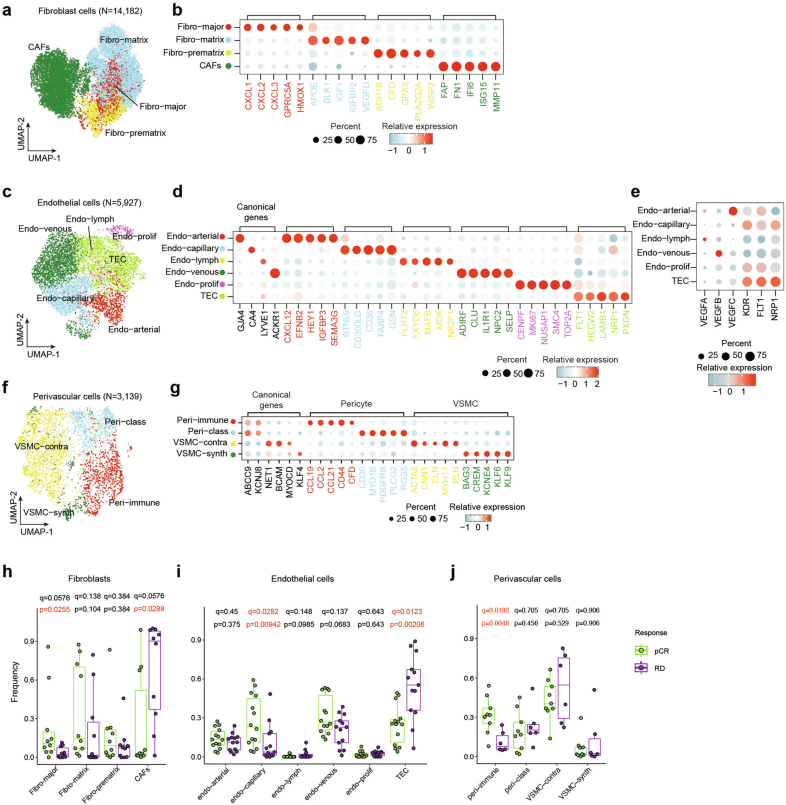
Stromal cell state and their associations with chemotherapy response, related to Fig. 4. **a**, UMAP plot of the integrated scRNA-seq data showing fibroblast cells (N = 14,182) colored by the fibroblast cell states. **b**, Dotplot showing the relative expression of marker genes of the fibroblast cell states. **c**, UMAP plot of the unintegrated scRNA-seq data showing endothelial cells (N = 5,927) colored by the endothelial cell states. **d**, Dotplot showing the relative expression of top marker genes for the endothelial cell states. **e**, Dotplot of marker genes associated with angiogenesis and tumor endothelial cells. **f**, UMAP plot of the unintegrated scRNA-seq data showing perivascular cells (N = 3,139) colored by the perivascular cell states. **g**, Dotplot showing the relative expression of marker genes of the perivascular cell states. **h**-**j**, Cell states composition differences for fibroblast cells (**h**), endothelial cells (**i**), and perivascular cells (**j**) in a comparison of the pCR and RD patients. In **h**-**j**, Wilcoxon rank-sum test was conducted with P-values adjusted by the Benjamini-Hochberg method, with significant values colored in red. Box plots show the median (center line), interquartile range (box limits), and 1.5x interquartile range (whiskers).

**Extended Data Fig. 7:**
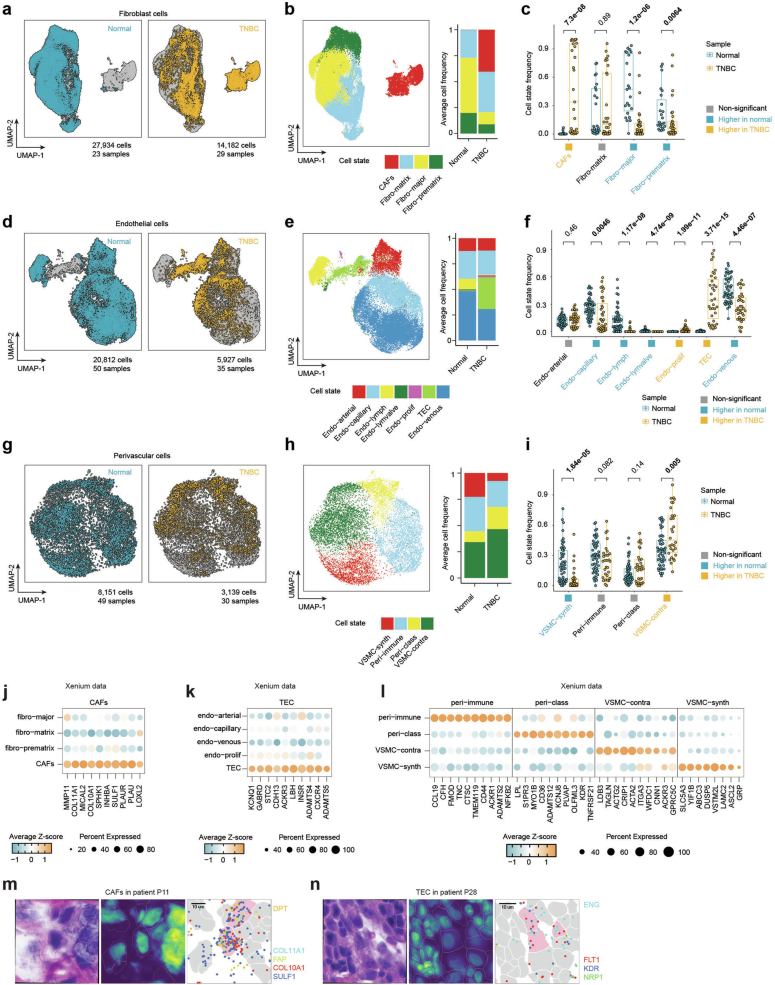
Stromal cell state differences between normal breast and TNBC, related to Fig. 4. **a**, UMAP plot showing the single cells of fibroblast cells in the integrated data of HBCA and TNBC. **b**, UMAP plot of the fibroblast cell states and stacked barplot showing the averaged cell state frequencies between HBCA and TNBC. **c**, Boxplot showing the compositional difference of fibroblast cell states between HBCA and TNBC. **d**, UMAP plot showing the single cells of endothelial cells in the integrated data of HBCA and TNBC. **e**, UMAP plot of the endothelial cell states and stacked barplot showing the averaged cell state frequencies between HBCA and TNBC. **f**, Boxplot showing the frequency difference of endothelial cell states between HBCA and TNBC. **g**, UMAP plot showing the single cells of perivascular cells in the integrated data of HBCA and TNBC. **h**, UMAP plot of the perivascular cell states and stacked barplot showing the averaged cell state frequencies between HBCA and TNBC. **i**, Boxplot showing the frequency differences of perivascular cell states between HBCA and TNBC. **j**,**k**,**l**, Xenium data showing the relative expression of the marker genes of CAFs (**j**), TECs (**k**), and 4 cell states in the perivascular cells (**l**). **m**,**n**, Xenium data showing in-situ expression of marker genes of CAFs (**m**) and TECs (**n**) in two representative patients (P11, P28). In **c**,**f**, and **i**, Wilcoxon rank-sum test was conducted with P-values adjusted by the Benjamini-Hochberg method. Box plots show the median (center line), interquartile range (box limits), and 1.5x interquartile range (whiskers).

Three stromal compartments including fibroblasts (*N* = 14,182), endothelial cells (*N *= 5,927) and perivascular cells (*N* = 3,139) were identified (Fig. 4e). Each cell type was further clustered into cell states on the basis of DEGs^34^ (Supplementary Table 8). Fibroblasts comprised fibro-major, fibro-matrix and fibro-prematrix cells, and cancer-associated fibroblasts (CAFs) with high expression of interferon-induced genes (*ISG15*, *IFI6*) and ECM-remodelling genes (*FAP*, *MMP11*, *FN1*) (Extended Data Fig. 6a,b). Endothelial cells comprised arterial, capillary, lymphatic, venous and proliferative (Endo-prolif) endothelial cells, and tumour endothelial cells (TECs) expressing angiogenesis-related genes (*HECW2*, *PLXND1*) and vascular endothelial growth factor receptors (*KDR*, *FLT1*, *NRP1*) (Extended Data Fig. 6c–e). Perivascular cells consisted of classical (*RGS5*, *PDGFRB*) and immune-signalling (*CCL2/19/21*) pericytes, as well as vascular smooth muscle cells (VSMCs) comprising differentiated contractile VSMCs (*NET1*,* ELN*) and dedifferentiated synthetic VSMCs (*KLF4/6/9*) (Extended Data Fig. 6f,g). Integration with HBCA revealed that CAFs, TECs and Endo-prolif were TNBC-specific, and that contractile VSMCs were higher in TNBC (*q* < 0.05, Wilcoxon), representing stromal reprogramming relative to normal breast tissue (Extended Data Fig. 7a–i). CAFs and TECs in TNBC were validated in situ by Xenium (Extended Data Fig. 7j–n). Comparison of stromal compositions between pCR and RD revealed that Peri-immune cells were enriched in pCR and TECs had higher abundance in RD (*q *< 0.05, Wilcoxon) (Fig. 4f–h and Extended Data Fig. 6h–j).

## Ecotypes and spatial niches

**Fig. 5:**
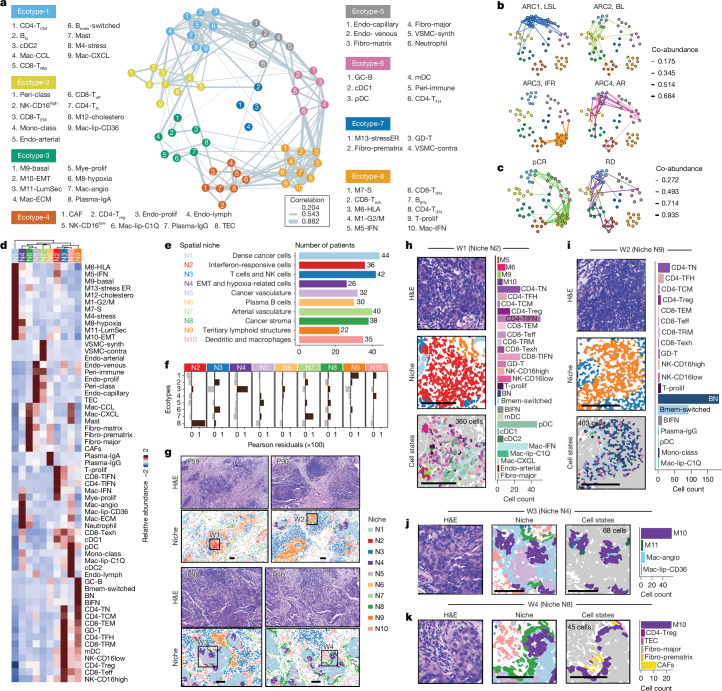
Ecotypes and spatial niches of cancer metaprograms and TME cell states. **a**, Network graph showing the co-occurrences of cell identities (that is, cancer-cell metaprograms and immune and stromal cell states) denoted by nodes. Edge width denotes Spearman correlation coefficient (SCC) of cellular percentages of two connected nodes among patients. **b**,**c**, Relative co-occurrence of cell identities across archetypes (**b**) and response groups (**c**). Edge width denotes the geometric mean of the averaged *z*-score cellular percentages of the two connected nodes. Only edges with non-zero widths are shown. **d**, Heatmap showing the relative abundance of cell identities across the ten spatial niches N1 to N10. The hierarchical clustering tree of niches (columns) was built on the basis of the CellTrek’s spatial colocalization result. **e**, Bar plot showing the number of patients in which the spatial niches were detected. **f**, Bar plot showing the Pearson residuals of the chi-squared test between the number of cells assigned to ecotypes and niches. **g**, Xenium data in two patient examples (P56, P90) showing H&E images, spatial niches and the locations of four expanded windows (W1–W4). **h**–**k**, Xenium data of the expanded windows W1–W4 showing H&E images, spatial niches and cell identities together with the bar plot of cell counts within the spatial niche examples for the niche N2 (**h**), N9 (**i**), N4 (**j**) and N8 (**k**). Scale bars, 100 μm.

**Extended Data Fig. 8:**
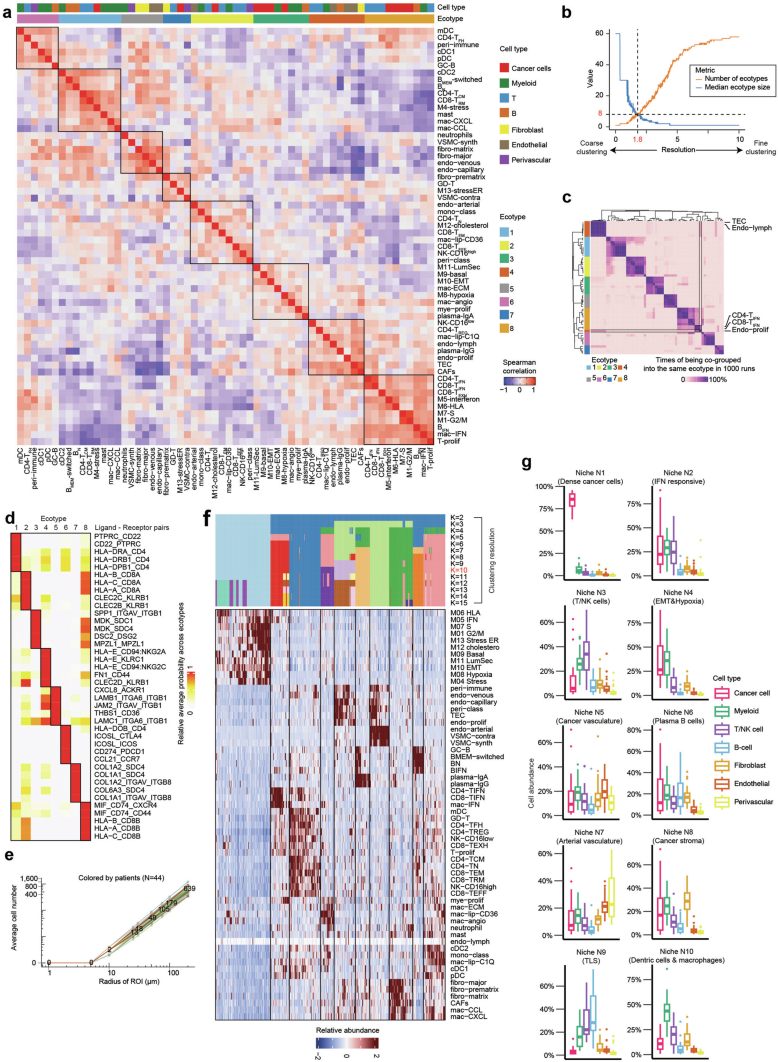
Identification of ecotypes, spatial niches, and cellular interactions, related to Fig. 5. **a**, Spearman correlation coefficients between the immune, stromal cell states and the cancer metaprograms based on the abundance among patients. **b**, Line plot showing number of ecotypes and the median size of ecotypes as a function of clustering resolution. **c**, Empirical probability of cell states and metaprograms being clustered into the same ecotype. **d**, Ligand-receptor interactions predicted within and between ecotypes using CellChat. **e**, Line plot showing the average cell number as a function of the radius of ROI in each patient. Lines are colored by patients (N = 44). The average cell number across patients is labelled for each selected ROI. **f**, Heatmap showing the cell percentage Z-scores of cell states across cell communities found by using all Xenium data (N = 44 patients). **g**, Boxplot showing the cell abundance of cell types within a niche. Each dot denotes a patient. Box plots show the median (center line), interquartile range (box limits), and 1.5x interquartile range (whiskers).

To identify multicellular communities, we constructed a co-occurrence network of cancer cells and TME cells using their cellular abundances within patients (Extended Data Fig. 8a). This network was partitioned into eight subnetworks, termed ‘Ecotypes’, each representing a collection of cancer-cell metaprograms and TME cell states that were simultaneously present or absent across patients (Fig. 5a, Extended Data Fig. 8b,c and Methods). These ecotypes suggested coordinated biological interactions. For example, Ecotype-3 comprised angiogenesis and/or ECM-related macrophage cell states (Mac-angio, Mac-ECM) and hypoxia and/or EMT-related cancer cells (M8-hypoxia, M9-basal, M10-EMT) with potential ligand–receptor interactions involved in cell adhesion and migration^53^ (Extended Data Fig. 8d). Ecotype-6 represented tertiary lymphoid structures (TLSs), comprising dendritic cells (cDC1, plasmacytoid dendritic cells, mature plasmacytoid dendritic cells), germinal centre cells and CD4-T_FH_, with predicted interactions through exhausted T cell receptors and HLA class II molecules^54^ (Extended Data Fig. 8d). Ecotype-8 contained interferon-associated immune cell states (CD4-T_IFN_, CD8-T_IFN_, Mac-IFN and B_IFN_) and cancer cells (M5-interferon, M6-HLA), expressing ligand–receptor genes including *MIF*, *CD74*, HLA class I genes and T cell receptors (Extended Data Fig. 8d).

**Extended Data Fig. 9:**
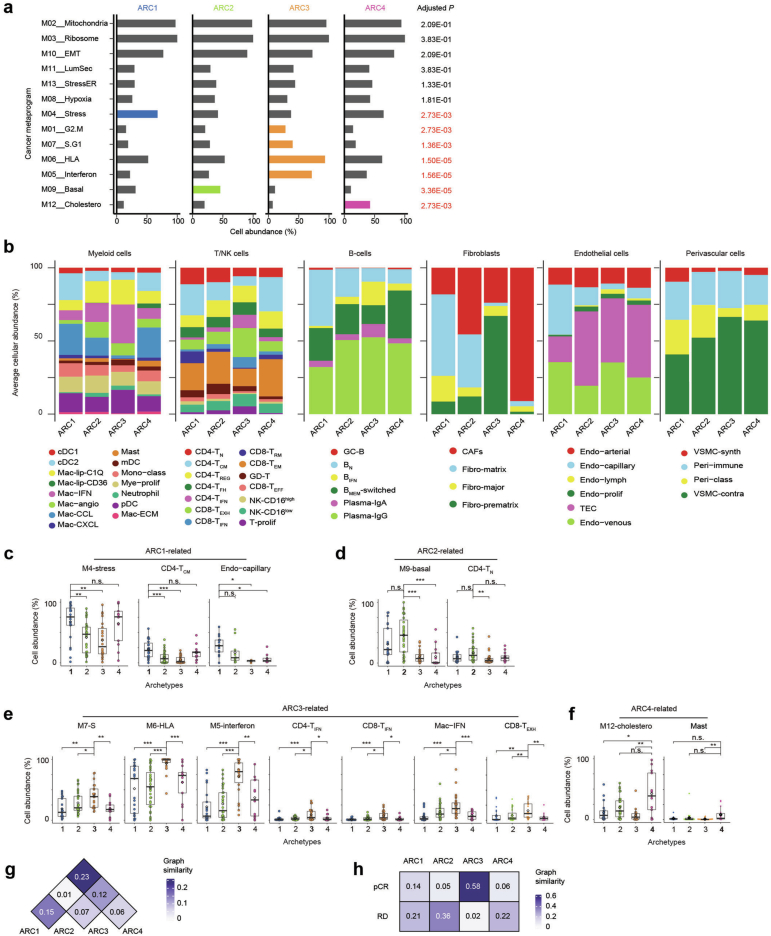
Cellular composition of the archetypes, related to Fig. 5. **a**, Barplot showing average cellular percentages of metaprograms in archetypes. **b**, Average cellular percentages of the cell states of myeloid cells, T/NK cells, B cells, fibroblast cells, endothelial cells, and perivascular cells in archetypes. **c**-**f**, Boxplots showing the cellular percentages of the cancer cell metaprograms and TME cell states that were enriched for ARC1 (**c**), ARC2 (**d**), ARC3 (**e**), and ARC4 (**f**). **g**, Jaccard similarity of co-occurrence graphs among archetypes. **h**, Jaccard similarity of co-occurrence graphs between archetypes and response groups. In **a**, Kruskal-Wallis test was conducted for all metaprogram with P-values adjusted by the Benjamini-Hochberg method. In **c**-**f**, Wilcoxon rank-sum test was conducted for all archetype comparison with *P*-values adjusted by the Benjamini-Hochberg method. n.s.: non-significant; *: Adjusted *P* < 0.05; **: Adjusted *P* < 0.01; ***: Adjusted *P* < 0.001; ****: Adjusted *P* < 0.0001. Box plots show the median (center line), interquartile range (box limits), and 1.5x interquartile range (whiskers).

Each archetype was enriched for different ecotypes (Fig. 5b). ARC1 was enriched for Ecotype-1 that included M4-stress and CD4-T_CM_ (Fig. 5b and Extended Data Fig. 9a–c). ARC2 was enriched for Ecotype-3 containing M9-basal (Fig. 5b and Extended Data Fig. 9a,b,d). ARC3 showed a high prevalence of the ‘hot’ TME-related Ecotype-8 (Fig. 5b and Extended Data Fig. 9a,b,e). The androgen receptor-related ARC4 was enriched for Ecotype-2, which included M12-cholesterol (Fig. 5b and Extended Data Fig. 9a,b,f). Consistent with the difference in cell-state composition across archetypes, the co-occurrence networks between archetypes showed low similarity (1–23%) (Extended Data Fig. 9g). Therefore, ecotypes reflect the cellular communities that were different across archetypes. Furthermore, the response groups showed distinct ecotype compositions. The pCR group was enriched for Ecotype-5, the TLS-like Ecotype-6 and Ecotype-8 containing the cell cycling-related metaprograms (M1-G2/M and M7-S), consistent with the anti-tumoural role of TLS^54^ and the mechanism of chemotherapeutic drugs (Fig. 5c). By contrast, the RD group was enriched for protumoural cellular communities including the cell migration-related Ecotype-3 and the immunosuppressive Ecotype-4 (for example, CD4-T_reg_, low cytotoxic NK-CD16^low^ cells, CAFs and TECs) (Fig. 5c). Finally, the network similarity analysis showed that the pCR group most closely related to ARC3 and the RD group was most similar to ARC2, supporting the clinical associations between archetypes and NAC responses (Extended Data Fig. 9h).

An important distinction is that cellular co-occurrences in single-cell data may reflect either proximal or distal cellular interactions. Therefore, ecotypes do not necessarily show spatial colocalization of cells. We next analysed Xenium data (*N* = 44 patients) to assess cellular colocalizations and identified ten spatial niches recurring across patients (Fig. 5d,e, Extended Data Fig. 8e–g and Methods). Several niches (N2, N4, N7, N9) corresponded closely to specific ecotypes, whereas the other niches (N3, N5, N6, N8, N10) included many ecotypes (Fig. 5f). Specifically, the interferon-associated Ecotype-8 cells were spatially colocalized in niche N2 (Fig. 5g,h). Ecotype-6 cells were colocalized within the TLS-like niche N9 (Fig. 5g,i). Ecotype-3 cells, including M10-EMT cancer cells and Mac-angio, were colocalized in niche N4 (Fig. 5g,j). By contrast, the cancer stroma niche N8 included M10-EMT cancer cells and stromal cell states (CAFs and TECs) that were from many ecotypes (Fig. 5g,k). Overall, we find that some ecotypes represent spatially localized clusters of cells, whereas other ecotypes indicate coordinated cellular communities that interact through more distal regions within the same tissues.

## Predicting NAC response

**Extended Data Fig. 10:**
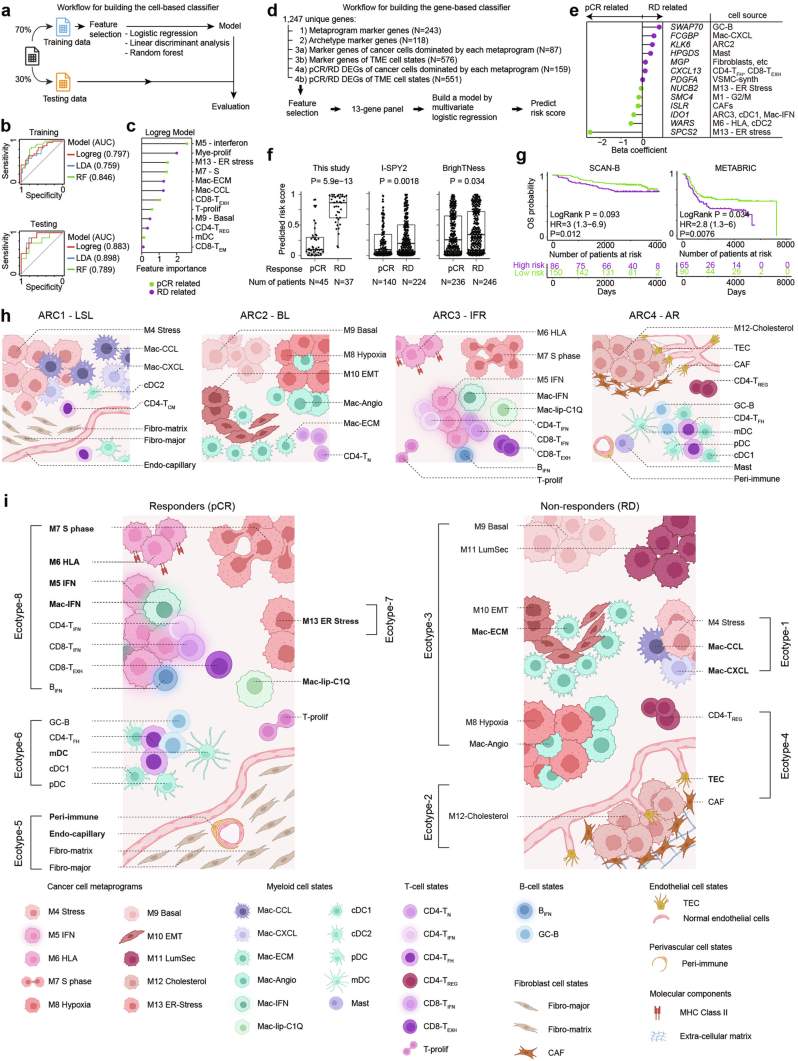
Classifiers for predicting NAC response and graphical summary. **a**, Workflow of building the cellular frequency-based classifier for predicting NAC response. **b**, Receiver operating characteristic curves (ROC) showing performance of classifiers on the training and testing datasets with AUC scores labelled. **c**, Lollipop plot showing the feature importance in the Log regression model. **d**, Workflow of building the gene-based multivariate logistic regression classifier for predicting the risk score of experiencing RD. **e**, Lollipop plot showing the beta-coefficient of the genes and the cell identify of the major expression source. **f**, Boxplots showing the predicted risk scores in this study and two external cohorts (I-SPY2 and BrighTNess) in a comparison of the pCR and RD patients. Wilcoxon rank-sum test was conducted. **g**, Association between the predicted risk score and the overall survival (OS) in two external cohorts (SCAN-B and METABRIC). HR: hazard ratio. **h**,**i**, Illustration showing the differences in cancer metaprograms and TME cell state compositions across the 4 archetypes (**h**) and the NAC response groups (**i**). In **i**, the bold text denotes the cell identities that showed significant differences in the pCR and RD patients, as reported in this study. Ecotypes were labelled with a subset of the cell states and cancer metaprograms. Box plots show the median (center line), interquartile range (box limits), and 1.5x interquartile range (whiskers). Panel **a** created in BioRender; Yan, Y. https://biorender.com/57adwi2. Panel **h** created in BioRender; Yan, Y. https://biorender.com/dg3yk8l, https://biorender.com/of02woy, https://biorender.com/k5ed9ul and https://biorender.com/gmnd6u2 (2026). Panel **i** created in BioRender; Yan, Y. https://biorender.com/h0zu10p (2026).

We developed a cell frequency-based machine learning model to predict NAC response (Extended Data Fig. 10a and Methods). Three models were developed: logistic regression (Logreg), linear discriminant analysis and random forest (Extended Data Fig. 10b). The ‘Logreg’ model showed the highest average performance (AUC = 0.84). The top features included three cancer-cell metaprograms and two myeloid cell states, indicating that NAC response was not dictated by either cancer cells or the TME cells and that the macrophage cells had a prominent role over other immune cell types (Extended Data Fig. 10c).

A small gene panel is often needed for rapid and practical clinical application using bulk tissue profiling. Therefore, we used the 1,247 response-related genes from the single-cell results and developed a multivariate Logreg model that used pseudo-bulk expression of 13 genes to predict risk of patients with TNBC experiencing RD (Extended Data Fig. 10d,e and Methods). Compared with the bulk sequencing-derived gene signatures^55^, the primary cellular sources of gene expression were known in our model (Supplementary Table 9). For example, *FCGBP* was expressed by Mac-CXCL cells, and *SPCS2* expression originated from the M13-ER stress cancer cells. For validation, we applied the model to two external cohorts of patients with TNBC (I-SPY2^45^ and BrighTNess^44^). The pCR and RD groups had significantly different risk scores in both cohorts (*P* < 0.05, Wilcoxon) (Extended Data Fig. 10f and Methods). Furthermore, we leveraged the overall survival data of two external TNBC cohorts METABRIC^40^ and SCAN-B^56^. The predicted risk score was significantly associated with overall survival in SCAN-B (*P* = 0.012, Wald test) and METABRIC (*P* = 0.0076, Wald test), and the high-risk patient group showed significantly shorter overall survival in METABRIC (*P* = 0.034, logRank test) (Extended Data Fig. 10g and Methods).

## Discussion

Here we report a large single-cell genomic study of early-stage TNBC and NAC response. On the basis of the cancer cells alone, we classified TNBC tumours into four patient-level archetypes. These archetypes reflect cancer-cell-intrinsic expression programs and differ from previous bulk tissue-derived subtypes that represent mixtures of cancer, stromal and immune cells^6–8^. Although it was previously unclear whether the ARC3 immunomodulatory group reflected the genes expressed in the cancer cells or immune cells^6,9,10^, our data clearly show that this signature is cancer-cell intrinsic. In our data, we found that ARC3 is associated with pCR, whereas ARC2 is associated with RD, indicating a potential clinical value of the archetypes. As TROP2 (encoded by *TACSTD2*) had uniformly high expression in the cancer cells across archetypes, the TROP2-directed antibody–drug conjugate (that is, the standard therapy for metastatic TNBC^5^) may also be effective in treating early-stage TNBC.

Overall, we identified 13 cancer-cell metaprograms and 49 immune and/or stromal cell states that formed 8 ecotypes, reflecting coordinated cellular communities associated with archetypes and NAC responses (Extended Data Fig. 10h,i). For example, our data identified a pCR-related ecotype comprising interferon signalling-related T cells (CD8-T_IFN_, CD4-T_IFN_), B cells (B_IFN_), myeloid cells (Mac-IFN) and cancer cells (M5-interferon). In addition, elevated expression of HLA class II genes in TNBC cancer cells (M6-HLA) is associated with pCR. Although interferon signalling and HLA genes are expected to be present in immune cells, the cancer-cell-intrinsic expressions of these genes in our data suggest a central role of cancer cells in responding or modulating immune signalling that relates to NAC response.

Although there has been an intense research focus on T/NK cells in TNBC^16,57^, our work also suggests a central role of macrophages in NAC response. Notably, none of the 14 T/NK cell states were significantly associated with NAC response, whereas most of the macrophage cell states (7 out of 8) were significantly different between pCR and RD, indicating dual roles in NAC response. The interferon signalling and C1q complement-associated macrophages (Mac-IFN and Mac-lip-C1Q) were more abundant in the patients showing pCR, whereas the angiogenesis-related and ECM-remodelling macrophages (Mac-angio and Mac-ECM) that occurred with the cancer cells expressing the M9-basal, M8-hypoxia and M10-EMT metaprograms were enriched in RD. These findings suggest that targeting specific macrophage subtypes could potentially provide new therapeutic opportunities in TNBC.

Because chemotherapeutic drugs have been the main component of all standard therapies for patients with TNBC, we developed a 13-gene-based panel from single-cell results to predict NAC response, which can potentially be implemented as a clinical diagnostic assay to facilitate oncologists and patients in making treatment decisions. The association of model predictions with chemotherapy response and overall survival across many public TNBC cohorts suggests a potential for future clinical translation, following further validation in prospective patients. We also constructed a cell-state frequency-based prediction model with a high predictive performance (AUC = 0.883). Although it remains expensive and technically challenging to perform routine clinical scRNA-seq using fresh core biopsy samples, experimental advances in efficient sample multiplexing^58^ and FFPE-compatible methods^59^ may facilitate future clinical implementation of scRNA-seq.

Comparison with normal breast tissue from HBCA revealed stark differences in the composition of immune cell types and states. Many of the immune cell states that were often assumed to be anti-tumoural (for example, CD4-T_IFN_, CD8-T_IFN_, B_IFN_, Mac-IFN) and protumoural (for example, CD4-T_reg_, CD8-T_exh_, Mac-angio) showed higher frequencies in TNBC. These findings highlight the complexity of protumorigenic and anti-tumorigenic immune cell roles in cancer progression and may provide extra marker genes that complement existing clinically used TIL-based gene panels^52^.

A notable limitation of our single-cell data is the relatively small number of stromal cells compared with the immune cells. This could reflect the technical bias of using core biopsy samples and microdroplet-based scRNA-seq platforms, which have been reported to enrich for smaller cell sizes (less than 50 µm) and rounder cells that do not undergo anoikis after dissociation (for example, immune cells)^60^. Future work should be directed towards chemo-immunotherapy treatment, because this has become the new standard of care for patients with early-stage TNBC^2^. Investigating longitudinal samples (pretreatment, on-treatment and posttreatment) may also provide new insights into the co-evolution of cancer cells and their TME, which may also hold value in predicting NAC responses or patient survival.

In closing, this study greatly improves our fundamental knowledge of the gene-expression programs of cancer cells and the diverse cell states of the TME in patients with TNBC. Our data lay the groundwork for developing new clinical diagnostic approaches to individualize therapy and targeting alternative immune cell types (for example, macrophages) to improve therapeutic responses of patients with TNBC.

## Methods

### Study participant details from the ARTEMIS Trial

The clinical trial protocol was reviewed and approved by The University of Texas MD Anderson Cancer Center Institutional Review Board and all participants provided written informed consent (NCT02276443; MDA no. 2014-0185). All study procedures performed were in accordance with ethical standards of the Institutional Review Board and with the 1964 Helsinki declaration and its later amendments or comparable ethical standards. TNBC was defined as breast cancer that was oestrogen receptor-negative (lower than 1%) or low oestrogen receptor-positive (1% to less than 10%), progesterone receptor-negative (less than 1%) or low progesterone receptor-positive (1% to less than 10%) and HER2-negative by immunohistochemistry and fluorescence in situ hybridization. Residual cancer burden (RCB) status after chemotherapy treatment was assessed by histopathology from the resected surgical specimen^61^. Chemotherapy response was classified into pCR (RCB-0) or RD (RCB-I, II or III) by RCB status. A subset of patients (*N* = 19) with suboptimal mid-treatment response were changed to targeted therapies before surgery and had no NAC response data available. The gene-expression data from patients with TNBC without NAC response data were used for the biological analysis (for example, cell type and cell state clustering) but were excluded from the analyses comparing the response groups. For each patient, 1–4 fresh tumour breast core needle biopsies were collected and transported to the laboratory in media for cell dissociation and frozen for spatial transcriptomic experiments.

### Experimental method details

#### scRNA-seq

The biopsies were placed in 10-cm sterile tissue culture dishes and were cut into pieces around 1 mm^3^, then digested in 1 ml of dissociation buffer (collagenase A (1 mg ml^−1^ working solution, Sigma no. 11088793001) dissolved in DMEM F12/HEPES media (Gibco no. 113300) and BSA fraction V solutions (Gibco no. 15260037) mixed at a 3:1 ratio, respectively). Cell suspensions were transferred into a 1.5-ml tube and rotated in a hybridization oven for 30 min at 37 °C. The cell pellet was collected by centrifuging at 500*g* for 5 min and then resuspended in 1 ml of trypsin (Corning no. 25053CI) before incubating in a rotating hybridization oven at 37 °C for 5 min. Next, 2 ml of DMEM containing 10% fetal bovine serum (Sigma no. F0926) was used for trypsin neutralization. The solution was then filtered through a 70-μm strainer (Falcon no. 352350) and then centrifuged at 500*g* for 5 min to collect the cell pellet. To remove red blood cells, the cell pellet was nutated at room temperature for 10 min in 10 ml of 1× MACS red blood cell lysis buffer (MACS no. 130-094-183). Then 10 ml of DMEM was added to stop red blood cell lysis and then centrifuged at 500*g* for 5 min. The resulting pellet was washed in 1 ml of cold DMEM and then resuspended in 100 µl of cold PBS (Sigma no. D8537) + 0.04% BSA solution (Ambion no. AM2616) and filtered through a 40-μm flowmi (Bel-Art no. h13680-0040) on ice until use. Before loading, cell viability and cell counts were quantified by Trypan blue staining with the Countless II FL automated cell counter (Thermo Fisher) and their concentration was adjusted to 300–1,000 cells per microlitre. Single-cell capture, barcoding and library preparation were performed by following the 10X Genomics Single Cell Chromium 3′ protocols (V2, CG00052; V3, CG000183; V3.1, CG000204). A detailed step-by-step description of this protocol is also publicly available at Protocols.IO 10.17504/protocols.io.t3geqjw.

#### Visium spatial transcriptomics experiments

Visium spatial transcriptomics experiments were performed using the Visium Platform (10X Genomics) following the manufacturer’s protocol with a minor modification based on the recommendations in the Tissue Optimization protocol (10X protocol no. CG000238). Specifically, fresh tumour biopsies were embedded in cryogenic (cryo) moulds with optimal cutting temperature (OCT) compound (Fisher nos. NC9542860, 1437365) on dry ice and stored at −80 °C until cryo-sectioning. Next 12-μm sections were prepared on a cryomicrotome (Cryostar NX70, Thermo Scientific) with chuck and blade temperatures set at −17 °C and −15 °C, respectively. Two core sections were placed within the capture area of the Visium spatial slide (10X Genomics PN-1000184). According to the manufacturer’s tissue optimization protocol, we determined that the optimal permeabilization time for breast tissue was 12 min. Sectioned slides were fixed and stained with H&E as described by the manufacturer (10X protocol no. CG000160). Images of H&E staining were captured on the Nikon Eclipse Ti2 system following imaging guidelines (10X protocol no. CG000241). The final libraries were constructed by following the user guide (10X protocol no. CG000239) and were sequenced on the Illumina next-generation sequencing system (NovoSeq6000) using the S2-100 flow cell.

#### Xenium in situ RNA experiments

Fresh frozen breast core biopsy tissues were embedded in cryomolds using OCT compound (Thermo Fisher Scientific no. 1437365) over dry ice and cryo-sectioned at 12 μm using a cryomicrotome (Leica, CM1950). Sections were placed within the capture area of the Xenium slides (10X Genomics, PN3000941). Tissue fixation and permeabilization were performed according to the 10X Genomics user guide (CG000581, revision D), with a modification extending the 37 °C incubation to 3 min for breast tissue samples. FFPE tissues were sectioned at 5 μm and placed on xenium slides by the pathology laboratory. Deparaffinization and decrosslinking were performed following the 10X Genomics user guide (CG000580, revision E), with a modification extending the nuclease-free water incubation time to 1 min to remove residual eosin from previously stained tissue before embedding. Both OCT and FFPE slides were subsequently proceeded according to the Xenium prime in situ gene expression with cell segmentation staining User Guide (CG000760, revision E). A custom 100-gene panel was also designed (Supplementary Table 4) and incorporated as an add-on to the Xenium Human 5K Pan Tissue & Pathways Panel. Post-Xenium H&E staining was conducted following the manufacturer’s protocol (10X Genomics user guide CG000613 revision A).

#### VisiumHD spatial transcriptomics experiments

Fresh frozen breast core biopsy tissues were embedded in OCT and cryo-sectioned at 12-μm thicknesses onto Nexterion H 3D hydrogel-coated slides (SCHOTT, no. 1800434). H&E staining and imaging were performed according to the VisiumHD Fresh Frozen Tissue Preparation Handbook (10X Genomics, CG000763, revision E), with a modification extending the 37 °C incubation step to 3 min for breast tissue samples. FFPE tissues were sectioned at 5-μm thicknesses and mounted on Nexterion H 3D hydrogel-coated slides. H&E staining, imaging and decrosslinking were conducted following the VisiumHD FFPE Tissue Preparation Handbook (10X Genomics, CG000684, CG000730). Post-Xenium prime slides were processed according to Post-Xenium in Situ Applications for VisiumHD (10X Genomics, CG000709, revision D). Autofluorescence Quenching Solution was removed following the Post-Xenium Analyzer H&E Staining Protocol (10X Genomics, CG000613, steps 1.1–1.3). H&E staining and imaging were performed as described in the VisiumHD FFPE Tissue Preparation Handbook (10X Genomics, CG000684). Tissue slides were imaged using a Nikon Eclipse Ti2 microscope in accordance with the VisiumHD Spatial Applications Imaging Guidelines (10X Genomics, CG000688). Subsequent library preparation and sequencing were performed following the VisiumHD Spatial Gene Expression Reagent Kits User Guide (10X Genomics, CG000685).

### Quantification and statistical analysis

#### Preprocessing of the scRNA-seq, Visium, VisiumHD and Xenium data

Sequencing reads of the scRNA-seq from the 10X Genomics Chromium protocol were demultiplexed using bcl2fastq (v.2.20). The scRNA-seq reads were aligned to the hg38 human genome reference using the CellRanger pipeline (v.3.1.0) with the default parameters. The Visium and VisiumHD reads were aligned to the hg38 human genome using the SpaceRanger pipeline (v.3.1.3) with the default parameters. For the VisiumHD processing, we used a bin size of 32 µm to combine features. According to the Xenium Onboard Analysis User Guide (CG000584, revision H), Xenium data and cell segmentation were generated by the xenium onboard analysis (v.3.3.0.1) using the xenium instrument software (v.3.3.0.0). The cell (or spot)-gene-expression matrix of each sample was normalized to 1 × 10^5^ and log_2_-scaled using Seurat (v.5)^62^.

#### Identification of aneuploid cells from scRNA data

We applied CopyKAT^36^ (v.1.0.8) to infer CNAs on the basis of gene-expression read counts from each single cell. Because our scRNA-seq data in most samples contained several cell types, we randomly picked roughly 800 cells from 1 sample from the HBCA dataset^34^ containing all the cell types and then mixed them with each TNBC sample to run CopyKAT. With the inferred CNA profiles, we further performed quality control in each sample using the Leiden-based method developed here and the Tirosh-like method modified from a previous study^37^. The cells from HBCA are all normal diploid cells, so they are used as the negative control to help identify any query cells with spurious CNA events.

The Leiden-based method determined aneuploidy if the query cells were not coclustered with the negative-control cells on the basis of the inferred CNAs profiles. The single-cell inferred CNAs profiles were subjected to principal component analysis (PCA). The top 30 PCA components were used to further perform dimensionality reduction to a two-dimensional space of UMAP. With the UMAP space coordinates of cells, a cell-to-cell similarity graph was built using ‘Rphenograph’ (v.0.99.1) at a granularity of using 50 nearest neighbours. On the basis of this similarity graph, the cell clusters were determined by using the function ‘leiden_find_partition’ of the ‘leidenbase’ package (v.0.1.8). To avoid under-clustering, clustering was performed stepwise with the clustering resolution, which started at 0.1 and increased by 0.1 per step. To avoid over-clustering, clustering stopped if either there were more than five clusters, or the resolution value reached two. As a result, a cell cluster was tentatively considered ‘aneuploid’ if the number and the percentage of the negative-control cells in the cluster were fewer than 10 and less than 1%, respectively.

The modified Tirosh-like method calculated the CNA score, which quantified the magnitude of genomic aberrations and the aneuploid correlation coefficient (ACC) that reflected whether a cell harboured the common CNAs profiles found in the sample. The CNA score of each cell was the square root of the sum of the squared CNA values of all genomic bins, where the CNA value was log_2_-scaled copy number ratio provided by CopyKAT. It was then rescaled against a value interval with the minimum and maximum specified as the 5% and 99.5% quantile of the CNA scores of the negative-control cells, respectively. To calculate ACC, the top 1% of cells with the highest CNA score were selected to compute the average CNA profile that represented the profile of the tentative aneuploid cells. A Pearson correlation coefficient between this profile and each single-cell inferred CNAs profile was calculated to yield ACC. The 99% quantiles of the CNA scores and ACCs of the negative-control cells were used as the threshold values for filtering. As a result, a single cell was tentatively considered ‘aneuploid’ if it had higher values than these two thresholds.

Taken together, each cell was finally designated as ‘aneuploid’ if it was classified as ‘aneuploid’ by both the Leiden-based and the Tirosh-like methods. Otherwise, the cell was determined as ‘non-aneuploid’.

#### Default scRNA-seq preliminary analysis using Seurat

The single-cell unique molecular identifier (UMI)-count data were normalized to a total UMI count of 1 × 10^5^ and log-scaled by using ‘NormalizeData’. *Z* scores of genes across single-cell were calculated using ‘ScaleData’ with total UMI counts regressed out to mitigate technical artefacts in downstream clustering. The top 2,000 high variable genes were determined by ‘FindVariableFeatures’. Linear dimension reduction of PCA was performed by ‘RunPCA’ with 300 principal components. With the top 200 PCA components, a cell-to-cell similarity neighbouring matrix was constructed by ‘FindNeighbors’, which further enabled a nonlinear dimension reduction of UMAP by ‘RunUMAP’ with the default parameters (for example, n.neighbors = 30). Louvain-based clustering by ‘FindClusters’ with various resolutions from 0.5 to 3 was performed to prepare for downstream annotation.

#### CCA-based integration

To mitigate potential batch-effect and patient-effect on data integration, we performed anchor-based canonical correlation analysis (CCA). In brief, we assigned single cells to arbitrary batches such that these batches had a balanced number of cells to facilitate performing CCA. The top 2,000 highly variable genes were determined by ‘FindVariableFeatures’ with the ‘vst’ method in each random batch data. These random batches were integrated by using the functions ‘SelectIntegrationFeatures’, ‘FindIntegrationAnchors’ and ‘IntegrateData’ with the default parameters.

#### DEG analysis

The DEGs using single-cell data were identified by applying the functions ‘FindMarkers’ and ‘FindAllMarkers’ of Seurat, which uses the Wilcoxon rank-sum test and corrects *P* values by the Bonferroni method. The DEGs using a pseudo-bulk count matrix were identified by using the functions ‘DESeqDataSetFromMatrix ‘, ‘DESeq’ and ‘results’ of the package ‘DESeq2’^63^ with the default testing parameters, which performed a Wald test and adjusted *P* values by means of the Benjamini–Hochberg procedure.

#### Filtering of scRNA-seq data and identifying main cell types

To remove low-quality cells, we retained cells that had at least 500 UMIs, at least 150 genes and a percentage of mitochondrial genes of less than 40%. To identify the main cell types, we computationally integrated the scRNA-seq data of all single cells of all samples using the CCA-based integration approach. Subsequently, our default scRNA-seq preliminary analysis using Seurat was performed. To identify the cell type for the resulting clusters, we identified the DEGs using ‘FindAllMarkers’ with the default parameters in a comparison of each cluster of being investigated and all the other clusters. By examining the top DEGs of each cluster, we determined the cell types on the basis of the canonical marker genes: epithelial (for example, *EPCAM*, *KRT6A/B*, *KRT7*, *KRT19*, *KRT8*), myeloid cells (for example, *SPI1*, *LYZ*, *APOC1*), T cells (for example, *CD3E/D*, *TRBC1*), NK cells (for example, *GNLY*, *NKG7*), B cells (for example, *MS4A1*, *JCHAIN*), fibroblast (for example, *LUM*, *DCN*, *COL1A1/2*), endothelial cells (for example, *FABP4*, *VWF*) and perivascular cells (for example, *RGS5*, *STEAP4*). Cell clusters with similar top DEGs were merged to form clusters at cell-type level.

Depending on the aneuploidy and cell-type identity, cells were separated into three groups: (1) cancer cells that were classified as aneuploid epithelial cells, (2) immune or stromal cells that were classified as non-aneuploid non-epithelial cells and (3) noisy or low-quality cells that were either aneuploid non-epithelial cells or non-aneuploid epithelial cells. Only the first two groups were kept for further processing. To remove doublets and multiplets, we enumerated each cell type and performed further clustering by applying the default Seurat clustering workflow. As previously described in ref. ^39^, we set the highly variable genes as the canonical markers of all cell types found in the HBCA dataset to identify doublets with the cancer cells. In each cell type, any clusters expressing DEGs of other cell types were considered doublets or multiplets and were removed from analysis. Last, cell clusters with mitochondria-related and noise-related top DEGs (for example, *NEAT1, MALAT1*) were also removed.

#### Calculating the frequency of CNAs in a patient cohort

To calculate the patient frequency of a CNA event (either gain or loss) along the genome, we first determined the CNA events present in each tumour. For each tumour, we computed the median of the log-scaled copy number ratio values of each genomic bin across single cells, which resulted in the consensus CNA profile of each tumour. For each consensus CNA profile, we used the mean value of all genomic bins as the ground state copy number and the s.d. as the deviation cut-off. Then we determined that a genomic bin had a CNA event of genomic gain if its copy number was greater than the mean + s.d., whereas it had a genomic loss if the copy number was smaller than the mean − s.d. Finally, the frequency of a CNA event in a genomic bin was determined as the number of consensus profiles having that event divided by the total number of consensus profiles.

#### Creating pseudo-bulk RNA-seq data

To create a pseudo-bulk RNA-seq data for a given group of single cells (for example, a patient or a cell type), the UMI counts of all single cells were summed for each gene. When there were many groups, we performed variance stabilizing transformation for normalization using the ‘vst’ function in DESeq2 (ref. ^63^) (v.1.34.0).

#### Performing NMF

Given a log_2_-scaled normalized UMI-count matrix *X*_*i*__*j*_, where $i\in \{1,2,\ldots ,G\}$, $j\in \{1,2,\ldots ,N\}$, *G* is the number of genes and *N* is the number of observations (for example, cells or patients), the gene expression of each gene among observations were centred to zero and then all the negative values were modified to zero. This processed matrix was then subjected to RcppML^64^ (v.0.3.7) to run non-negative matrix factorization (NMF). The parameter ‘tol’ controlling tolerance was set to 1 × 10^−5^ to allow sufficient iterations and achieve a high approximation. With a fixed number of factors (that is, *K*), NMF resulted in two matrices: *W*_*ik*_ showing genes-to-factors contributions and *H*_*kj*_ showing factors-to-observations contributions, where $k\in \{1,2,\ldots ,K\}$. NMF was not performed when *K* exceeded *N*.

#### Determining marker genes of a NMF factor

Marker genes of each factor were determined on the basis of the gene-to-factor contribution matrix *W*_*ik*_ of NMF^64^. For a factor indexed at *k*, genes were sorted in a decreasing order on the basis of the values in the column W_*k_. Then each gene *g* was enumerated to be added to the set of marker genes until the specific value *W*_*gk*_ was no longer the maximum of the row *W*_g*_, as previously described in ref. ^65^. To accommodate genes possibly shared by several factors, we tolerated at most two more genes being added when the value *W*_*gk*_ was not the maximum. This procedure was later used to define marker genes for archetypes and metaprograms.

#### Identifying archetypes

In contrast to the conventional bulk RNA-seq that reflected signals from the intermixed cell types, we created a pseudo-bulk RNA-seq gene-expression matrix using cancer cells only for each patient and then performed NMF on all pseudo-bulk data to identify archetypes. In detail, the top 75% of genes in terms of the mean and variance of gene expressions were retained. This filtered pseudo-bulk gene-expression matrix was subjected to NMF using several numbers of factors *K* ranging from 2 to 10. Each factor denoted an archetype. The parameter ‘L1’ controlling sparsity of NMF results was set as 0.05. To determine the most possible number of archetypes in the dataset, we used the function ‘fviz_nbclust’ of ‘factoextra’ (v.1.0.7) to estimate the silhouette scores that compared the patient-to-patient Spearman correlation matrix of gene expressions and the archetype assignments resulting from the series of *K* values. Last, the resulting matrix *H*_*kj*_ was used to assign each patient *j* by its highest factor, representing the designated archetype. The gene signature of an archetype was determined as the shared genes between the marker genes of each archetype (that is, a NMF factor) and the DEGs of each archetype that showed a fold change of 2 or less and Benjamini–Hochberg-adjusted *P* < 0.05.

#### Measuring archetype specificity

For each sample, the log_2_ ratio between the two top archetypes and the Tau score^66^ were computed using the NMF loading matrix *H* for scRNA-seq data and using the overall expression levels of archetype gene signatures for Xenium data. To determine the thresholds for each metric, we used the resulting values in all samples and fitted two-component Gaussian mixture model using the ‘normalmixEM’ function in the mixtools package with default parameters. The mean of the lower component was used as the threshold. Last, if both the Tau score and log_2_ ratio were below the thresholds, the archetype specificity was ‘ambiguous’ in the sample; otherwise, it was ‘dominant’.

#### Identifying metaprograms of cancer cells

In brief, we enumerated each sample and performed NMF on single cancer cells to identify the transcriptional programs that were heterogeneously expressed. The transcriptional programs showing similar marker genes in many samples were merged to derive metaprograms. In detail, we excluded genes having an average log_2_-scaled normalized expression no greater than 0.05 and samples having fewer than 20 cancer cells. The gene-expression matrix of each sample was subjected to NMF using a series of number of factors (that is, $K\in \{2,4,\ldots ,30\}$). Each NMF factor represented a transcriptional program, and the marker genes of the programs were determined. Jaccard similarities between all detected programs were computed on the basis of their marker genes. A program was considered noisy and excluded if it had a Jaccard similarity less than 0.25 with its most similar program other than itself.

To find the proper clusters of the programs to form metaprograms, we repeatedly applied the following procedure of clustering, cutting, grouping, filtering and merging. Hierarchical clustering was performed on the Jaccard similarity matrix with the ‘ward.D2’ linkage method by use of the function ‘hclust’. We cut the tree into several groups of programs with a series of number of cuts (that is, $C\in \{10,12,\ldots ,30\}$) using the R function ‘cutree’. On the basis of the grouping result of the maximum cut *C* = 30, we computed the intra-group and inter-group Jaccard similarity scores. We filtered out groups having an intra-group Jaccard similarity score less than 0.1. We merged groups if they had mutual inter-group Jaccard similarity scores greater than 0.2 and were found in the same group resulting from a smaller value of cut. Each round of the procedure resulted in tentative metaprograms and was repeatedly performed until there was no further change. Last, we averaged the gene-to-factor contribution values of the NMF factors assigned to each metaprogram to make the gene-to-metaprogram contribution matrix, which was used to determine the marker genes of each metaprogram (that is, a NMF factor). Metaprograms characterized by genes associated with technical noise were excluded.

#### Testing the association of archetype and response

We first built the contingency table of the archetype assignments and NAC responses of the patients. Then a chi-squared test was performed using the R function ‘chisq.test’, which provided a chi-squared test statistic, degrees of freedom and a *P* value to evaluate significance. This test also provided the Pearson residual showing the direction of association.

#### CNA inference in Visium data

To find the spatial transcriptomic spots having a high abundance of cancer cells in each sample, we performed the ‘Tirosh-based’ approach modified from previous work in ref. ^37^. In each sample, we performed CopyKAT^36^ resulting in a CNA profile for each spot. The CNA profiles of all spots were averaged to represent the reference CNA profile of the malignant cell population. For each spot, the squared CNA values of all genomic bins were averaged to represent the aberration score. A Pearson correlation between the CNA profile of a spot and the reference CNA profile was calculated to estimate whether the spot had the common CNA events detected in the sample. Using the inferred CNA profiles of all spots, we then ran the functions ‘findSuggestedK’ and ‘findClusters’ of the package Copykit^67^ to determine spot clusters harbouring similar CNA profiles. The cluster of spots with an average aberration score >0.005, an average correlation >0.3 and the known CNA events (for example, chromosome 8 gain) were classified as ‘abnormal’: otherwise, they were ‘normal’. Furthermore, depending on the average aberration score, the cancer-cell abundance of a ‘abnormal’ spot cluster was further classified as ‘high’ if it was greater than 0.045, ‘medium’ if it was in the range of 0.02–0.045 or ‘low’ if it was less than 0.02.

#### Identifying cell types and cell states in Xenium data

Identification of cell types and cell states used the Label Transfer approach from Seurat^62^, which integrated a reference scRNA-seq data with a query Xenium data and projected the cell identities from the scRNA data to the Xenium data. First, we created a reference scRNA-seq data by randomly down-sampling at most 1,000 cells for each cancer-cell metaprogram and TME cell state and selecting genes in the Xenium gene panel. To improve computational robustness, we performed ‘label transfer’ using a tenfold cross validation approach. We split the genes evenly into ten subsets, such that each subset maintained the same statistical distribution of gene expression as the whole gene set. Then label transfer was performed consecutively ten times with nine gene subsets used each time. Last, the consensus transferred label was used as the cell identity for each cell. In detail, we performed label transfer for each Xenium sample to determine cell types while we enumerated each cell type of the immune and stromal cell types to integrate all Xenium data to perform label transfer, which resulted in cell states. As doublets or low-quality cells did not receive the same votes for cell identities among the ten label transfer results, we required a cell to have the same transferred cell type in all the ten votes. The threshold of votes for a transferred cell state was the maximum votes, such that the total probe count, the detected gene count and the label transfer probability of cells were no longer significantly different from the cells with ten votes (Wilcoxon test, Benjamini–Hochberg-adjusted *P* < 0.05). If this vote threshold was less than five, it was manually designated as five. Doublets or low-quality cells were removed from this analysis.

#### Identifying cancer-cell bins in VisiumHD data

VisiumHD with a bin size of 32 µm was used to increase gene counts for cells. The bins with total UMI counts fewer than 10 and a gene number fewer than 30 were removed from analysis. VisiumHD data were subject to the same default scRNA-seq preliminary analysis using Seurat. The aneuploid bins were identified using the same pipeline of scRNA-seq aneuploid cell identification. Cell types of bins were identified by the Robust Cell Type Decomposition method in the package spacexr^68^ (v.2.2.1). As the reference data to run Robust Cell Type Decomposition, we first leveraged the HBCA scRNA-seq data and then used the TNBC scRNA-seq data from this study. The bins that were ‘aneuploid’ and were predicted as ‘epithelial cells’ and ‘cancer cells’ were finally designated as the cancer-cell bins.

#### Gene signature levels in spatial transcriptomics

We used AUCell^69^ (v.1.16.0) to quantify the overall expression level of a gene signature (for example, archetypes) in the spatial transcriptomics data. In brief, we used the function ‘AUCell_buildRankings’ with the default parameters to build the gene-expression ranking matrix of all genes across all spots. We applied the function ‘AUCell_calcAUC’ and specified the parameter ‘aucMaxRank’ to use the top 20% of the genes in the ranking matrix, which resulted in the ‘AUCell value’ estimating enrichment level of a gene signature in spots.

#### Calculating module scores of a metaprogram in single cancer cells

For each metaprogram, module scores of single cells were computed using the expression of marker genes while randomly selecting genes with comparable expression levels as the control^37^. Specifically, we applied the function ‘AddModuleScore’ of Seurat^62^ on all cancer cells from all samples to calculate the module scores.

#### Assigning cell cycle phase for single cells

We applied the function ‘CellCycleScoring’ of Seurat with the cell cycle phase-specific genes provided by Seurat to all the cancer cells to assign the cell cycling phase.

#### Integrating TME of TNBC and the normal breast tissues

We randomly selected the scRNA-seq data of 50 disease-free individuals from the HBCA dataset^34^. For each non-cancer cell type (that is, myeloid cells, T/NK cells, B cells, fibroblast, endothelial cells and perivascular cells), we used CCA-based integration approach to integrate the single cells from patients with TNBC and normal breast tissue data from HBCA.

#### Immune and stromal cell states in scRNA-seq data

For the immune (myeloid, T/NK and B cells) and stromal (fibroblast, endothelial and perivascular cells) cell compartments, we performed further subclustering for each cell type using the following procedure. Subclustering was performed by using the function ‘FindClusters’ with a series of clustering resolution ranging from 0.5 to 3. The DEGs of clusters were identified by using the function ‘FindAllMarkers’. Clusters showing similar DEGs were merged to avoid over-clustering. We also used the package ‘clustree’^70^ (v.0.4.4) and referred to the results of integrating with HBCA to guide choosing the proper resolution and merging cell clusters. Cell subclusters expressing canonical marker genes from other lineages were considered doublets and removed from analysis.

#### Single-cell UMAP visualization of cells in scRNA-seq data

To facilitate single-cell UMAP visualization of all cells, all cancer cells, all immune cells or all stromal cells, we applied the default single-cell analysis workflow with the following adjustments of parameters. For all cells, all cancer cells and all immune cells, we simply merged the single-cell data instead of applying CCA-based integration. For all stromal cells, we used the integrated data. Then we re-identified 5,000 high variable genes and reperformed PCA with 500 components. The top 300 PCA components were used. Finally, to regenerate the UMAPs for all cells and all cancer cells, we did not adjust parameters of ‘RunUMAP’. We changed the parameters to n.neighbors = 20, min.dist = 1 and spread = 0.75 for all immune cells, and changed to min.dist = 3 and spread = 1.5 for all stromal cells.

#### Cell frequency of the metaprograms and TME cell states

The cell frequency of a metaprogram in a sample was calculated as the ratio between the number of cancer cells having a module score no smaller than 0.1 and the total number of cancer cells. In the sample without cancer cells, the cellular frequencies of metaprograms were assigned as ‘not detected’ (that is, the ‘NA’ value) instead of 0. The cell frequency of a TME cell state in a sample was computed as the fraction of the cell state in the corresponding cell type. This fraction was also designated as not detected if the corresponding cell type was not found in the sample. This calculation procedure was applied to scRNA-seq and Xenium data. Comparison of cell frequencies between two groups was subject to the Wilcoxon signed-rank test providing exact *P* values.

#### Co-occurrence of cancer cells and TME cells

The co-occurrence network was based on Spearman correlation coefficients (SCCs) of TME cell states and cancer-cell metaprograms using their cell frequencies among samples. We used the R function ‘cor’ with the parameter ‘use’ specified as ‘pairwise.complete.obs’ to compute SCCs and statistical significance. We then used the package ‘igraph’ (v.1.5.0.1) to convert the SCC matrix to an undirected weighted network by considering TME cell states and metaprograms as nodes and SCC scores as edge widths. Only correlations with *P *< 0.05 were kept for further analysis and visualization.

#### Determining ecotypes of cancer metaprograms and TME cell states

On the basis of the co-occurrence network, we performed community detection to determine the subnetworks to define the ecotypes. We first filtered out all edges having a negative SCC score. To find a proper clustering resolution to apply the function ‘cluster_louvain’ of ‘igraph’, we evaluated the number of ecotypes and the median size of ecotypes that each was a function of clustering resolution values ranging from 0.01 to 10. As the clustering resolution increased, the number of ecotypes increased and the median size of ecotypes decreased. We determined that the optimal clustering resolution value was where the two functions crossed. To alleviate the computational stochasticity of ‘cluster_louvain’, we performed clustering 1,000 times using the optimal clustering resolution value and built an instance matrix recording the number of times that any two nodes were clustered into the same ecotype. Finally, we applied the function ‘infomap.community’ of ‘igraph’ on this instance matrix to determine the communities that defined the ecotypes.

#### Visualizing the co-occurrence network

Edges with a SCC score less than 0.2 were considered spurious and were filtered out. We then heuristically changed the parameter ‘weights’ of the igraph’s function ‘layout_with_fr’ to get a graph layout to visually separate the ecotypes, while the original SCCs between any two nodes were reserved. Specifically, if an edge connected two nodes that were assigned to the same ecotype, the ‘weights’ parameter was set as 18; otherwise, it was 1. This layout was used for all graph visualizations of the co-occurrence network.

#### Comparing the co-occurrence network across patient categories

To facilitate comparing the co-occurrence network across patient categories (for example, pCR and RD), we computed and visualized the relative copresence (RCOP) scores among the TME cell states and the cancer metaprograms for each patient category. In detail, using the cell frequency matrix *F*_*ij*_ where *i* denoted the TME cell states and the cancer metaprograms and *j* denoted the patients, we calculated a *z*-score for each row to yield a relative cell frequency matrix *Z*_*ij*_. Then for each patient category, we applied the following procedure to compute a category specific RCOP score matrix. The matrix *Z*_*ij*_ was subset by using the patients assigned to the specific category. The row averages of this subset matrix were calculated to yield a vector **z**_*i*_. To calculate the RCOP score between a pair of TME cell states and cancer metaprograms that were indexed at *a* and *b*, we computed the geometric average ${{\rm{e}}}^{[{\rm{ln}}[A]+{\rm{ln}}[B]]/2}$, where *A *= *z*_*a*_ and *B *= *z*_*b*_. RCOP was assigned zero if either *A* or *B* was negative. Last, we obtained a RCOP score matrix for each specific patient category, which was visualized on the graph of the co-occurrence network. To quantify the similarity between the graphs of two patient categories, we computed the Jaccard similarity of two graphs on the basis of their edges with a non-zero RCOP score.

#### Ligand–receptor analysis

Ligand–receptor analysis was performed using the CellChat package^71^. For each cancer-cell metaprogram and TME cell state, we randomly down-sampled to about 20% of the cells. We then performed the default CellChat workflow using the functions ‘identifyOverExpressedGenes’, ‘identifyOverExpressedInteractions’, ‘computeCommunProb’, ‘computeCommunProbPathway’ and ‘aggregateNet’. We then used the function ‘netAnalysis_contribution_allLR’ to obtain the probabilities of all ligand–receptor pairs to compute the intra-ecotype and inter-ecotype signals, which were used for the downstream visualization.

#### Spatial niche analysis in Xenium data

Spatial niches were identified on the basis of the cell composition within the regions surrounding single cells. In detail, we enumerated the Xenium data of each patient and computed the percentages of all cancer-cell metaprograms and TME cell states in the regions having a radius of 30 µm with single cells as the centres. Each cancer cell was labelled as the highest metaprogram with a module score greater than 0.1. This step generated a new single-cell data matrix representing the local cell composition of each cell in each sample. Then we clustered each cell composition matrix to identify niches in each sample. To speed up this clustering step, we applied the ‘SketchData’ function of the Seurat package^62^ to run the clustering using ‘FindNeighbors’ on 2 × 10^5^ cells in each patient and applied the ‘ProjectData’ function to provide the clusters of all cells in the sample. To avoid over-clustering, we computed the average cell percentages of these clusters that were subjected to the ConsensusClusterPlus package^72^ to merge similar clusters. Last, after we computed the average cell percentages for these robust clusters in each patient, we used all these clusters found in all patients to run clustering using the ConsensusClusterPlus package, which resulted in the spatial niches that were present in several samples. Each cell was assigned to a spatial niche. The average cell abundance across spatial niches was used for biological annotation and visualization. In addition, we applied the function ‘scoloc’ of the package CellTrek (v.0.0.94)^73^ with the parameter ‘use_method’ set to ‘DT’ to compute the spatial colocalization odds ratio matrix among cell states that were averaged on the basis of the niches to run hierarchical clustering and determine the spatial relationship between niches.

#### Selecting patients and treatment arms in public cohort datasets

To obtain validation datasets that were comparable to this study, we selected the pretreatment bulk sequencing-based gene-expression datasets of patients with TNBC from four public clinical trials including SCAN-B^56^ and I-SPY2^45^, as well as BrighTNess^44^ in which patients received neoadjuvant therapy and METABRIC^40^ in which patients received adjuvant therapy. First, we subset the patients who were either labelled as TNBC or were negative for the expression of endoplasmic reticulum, PR and HER2 as recorded in the original clinical metadata. For METABRIC, all the treatment arms containing chemotherapy were used in all the analysis. For SCAN-B, only the chemotherapy arm was used in all the analysis. For I-SPY2 and BrighTNess, all the treatment arms containing chemotherapy were used for the application of the 13-gene model.

#### Predicting the archetypes in external TNBC cohort data

The bulk RNA-seq data matrix of an external cohort was first subjected to same rescaling step of the section ‘Performing NMF’. This rescaled matrix was then combined with the NMF input data matrix of our samples, which was an internal control. We used this combined matrix to run the same NMF procedure using the RcppML package with a rank of 4. By leveraging the archetype designation of our TNBC data, we renamed each resulting cluster to the archetype showing the highest matches. Accuracy of the prediction in each external cohort was assessed by the percentage of the predicted archetypes that matched the established archetypes in our TNBC samples.

#### Survival analysis using the Cox proportional-hazards model

To assess how numeric variables (for example, age, module score of a gene signature and risk score predicted by a model) were associated with overall survival of patients in a cohort, we applied the Cox proportional-hazards model to the variables of interest and the overall survival data. To compute a gene signature, the overall expressions in patients were computed using the function ‘gsva’ of the package ‘GSVA’^74^ (v.1.42) with the parameter ‘method’ set as ‘ssgsea’, and then the *z*-score was computed. We used the function ‘coxph’ of the package ‘survival’^75^ (v.3.3) to construct the Cox proportional-hazards model. The hazard ratios and *P* values of the Wald test were extracted from the modelling result and visualized using the function ‘ggforest’ in the package ‘survminer’ (v.0.4.9).

#### Building the cell-state-based classifier to predict NAC response

Considering the TME cell states and cancer-cell metaprograms as features, we excluded features detected in no more than ten samples. Samples without response data were excluded from this analysis. We randomly split samples into 70% for training and 30% for testing. Because the number of features was greater than the number of training samples, we performed a preliminary feature selection by univariate logistic regression and retained the features with a *P* value less than 0.1. With these informative features, we used the package ‘caret’^76^ (v.6.0-90) to train the following three models: logistic regression, linear discriminant analysis and random forest. During the training, the fivefold cross validation was repeated five times. The performance of each model was evaluated on the testing dataset and visualized by using the ‘pROC’ package^77^ (v.1.18). The feature importance values were computed by running caret’s function ‘varImp’ on each model.

#### Building the gene-based classifier to predict NAC response

We started with a pool of candidate genes that were associated with chemotherapy response using: (1) the marker genes of the cancer metaprograms and archetypes, (2) the DEGs across the TME cell states and the cancer cells labelled by the most dominant metaprogram and (3) the DEGs of the TME cell states and the cancer cells labelled by the most dominant metaprogram in a comparison of pCR and RD. Using the pseudo-bulk RNA-seq data derived from all cells, we enumerated each gene and applied univariate logistic regression to find the genes significantly predicting chemotherapy response (*P* < 0.01). As a result, 13 genes were identified and then were used to build a multivariate logistic regression model to yield a risk score for predicting the likelihood of a patient having RD.

#### The gene-based classifier and overall survival

The METABRIC^40^ and SCAN-B^56^ cohorts were used for this survival analysis. For each gene, we first rescaled its expressions among the patients in the external cohort into the value range of the expressions in our pseudo-bulk data, to mitigate possible batch effects. Then patients were stratified into high-risk and low-risk on the basis of the mean of the predicted risk scores. We used the function ‘survfit’ of the package ‘survival’^75^ (v.3.3) to compare the difference of the overall survival curves between the two groups. The *P* value of the LogRank test was extracted by the function ‘surv_pvalue’. The Kaplan–Meier plot was visualized by ‘ggsurvplot’ of the package ‘survminer’ (v.0.4.9).

#### Gene signatures of the ‘Vanderbilt’ expression subtypes

Because the original study of ‘Vanderbilt’ subtypes^9^ did not directly provide the gene signatures for each subtype, we downloaded and processed their DEG results. In detail, we retained the DEGs that were labelled as ‘upregulated’ in each subtype and had an adjusted *P* value of less than 0.05. The original gene alias names were converted to the updated gene symbols using the function ‘alias2Symbol’ of limma^78^ (v.3.50.3). The resulting gene signatures are available at Zenodo (10.5281/zenodo.10846115)^79^.

### Reporting summary

Further information on research design is available in the Nature Portfolio Reporting Summary linked to this article.

## Online content

Any methods, additional references, Nature Portfolio reporting summaries, source data, extended data, supplementary information, acknowledgements, peer review information; details of author contributions and competing interests; and statements of data and code availability are available at 10.1038/s41586-026-10469-9.

## Supplementary information

Supplementary Tables Supplementary Tables 1–9.

Reporting Summary

## Data Availability

The data are available from the Sequence Read Archive at PRJNA1041570 and from CZI CellXGene at https://cellxgene.cziscience.com/collections/0a117356-ecaa-4b82-a454-c15a4c9ec507.

## Code availability

Code is available from GitHub at https://github.com/navinlabcode/tnbc-chemo.
