# Supplementary material for: Ecotypes of triple-negative breast cancer in response to chemotherapy
Source: Nature. 2026 May 13;654(8120):1088–97. doi: 10.1038/s41586-026-10469-9 (PMC13293894; doi:10.1038/s41586-026-10469-9)
Supplement: Supplementary file 1 — Supplementary Tables 1–9. [file 41586_2026_10469_MOESM1_ESM.zip › 2024-08-17057C-s1/Cover_for_supp_tables.docx]

**Ecotypes of Triple-Negative Breast Cancer in Response to Chemotherapy**

Yan, Lin, Kumar, *et al.*

**Supplementary Information**

**Table of Contents**

|  |  |
| --- | --- |
| Supplementary Table 1 | Clinical information of the patients. |
| Supplementary Table 2 | Metrics of the scRNA-seq data. |
| Supplementary Table 3 | Metrics of the Xenium data. |
| Supplementary Table 4 | The custom panel of 100 additional genes for Xenium. |
| Supplementary Table 5 | DESeq2 results showing the statistically significant DEGs in each archetype. |
| Supplementary Table 6 | Gene signatures of archetypes. |
| Supplementary Table 7 | Marker genes of the cancer cell metaprograms. |
| Supplementary Table 8 | Top 20 DEGs of cell states in the TME cell types. |
| Supplementary Table 9 | The 13-gene classifier model. |
